# Positioning the femoral bone socket and the tibial bone tunnel using a rectangular retro-dilator in anterior cruciate ligament reconstruction

**DOI:** 10.1371/journal.pone.0215778

**Published:** 2019-05-02

**Authors:** Hiroteru Hayashi, Daisaburo Kurosaka, Mitsuru Saito, Ryo Ikeda, Daisuke Kubota, Tomohiro Kayama, Takashi Hyakutake, Keishi Marumo

**Affiliations:** Department of Orthopaedic Surgery, The Jikei University School of Medicine, Tokyo, Japan; Rothman Institute, UNITED STATES

## Abstract

**Purpose:**

The purpose of this study was to evaluate the positions of femoral bone sockets and tibial bone tunnels made with the rectangular retro-dilator (RRD), which we manufactured for anterior cruciate ligament reconstruction (ACLR) with a bone-patella tendon-bone (BPTB) graft which is fixed into the rectangular bone socket and tunnel made at anatomical ACL insertion sites.

**Methods:**

42 patients who had undergone ACLR with BPTB using the RRD were evaluated to assess bone socket and tunnel positions by the quadrant method and Magnussen classification using three-dimensional (3-D) CT. Intra-operative complications were also investigated in all patients.

**Results:**

3-D CT of the operated knee joints using the RRD showed that the bone socket and tunnel were placed in anatomical positions. In the quadrant method, the mean position of the femoral bone socket aperture was located at 22.0 ± 4.2% along the Blumensaat’s line, and 37.4 ± 7.2% across the posterior condylar rim. The mean positions of the tibial bone tunnel aperture were 37.7 ± 5.2% and 46.1 ± 2.2% antero-posteriorly and medio-laterally, respectively. In addition, according to the Magnussen classification, 39 cases were evaluated as type 1, and almost all were located behind the lateral intercondylar ridge (also known as the resident’s ridge). 3 cases were classified as type 2, which overlapped with the resident’s ridge. A partial fracture of BPTB bone fragment was observed in 2 patients, but no serious complications including neurovascular injury were observed.

**Conclusion:**

The study indicates that the use of RRD achieves a safe anatomical reconstruction of the ACL.

## Introduction

In recent years, there have been reports that the locations of the femoral bone socket and the tibial bone tunnel aperture affect the clinical outcome in ACLR, and its importance has been increasingly recognized [1,2]. Until recently, the trans-tibial technique was mainstream, but there have also been reports of the antero-medial portal technique and also the outside-in technique, which is a method to create a tunnel from the outside. The outside-in retrograde drilling technique in which a guide pin is inserted from the outside, but the tunnel is created from the within the joint with a retrograde drill has also demonstrated good clinical results [3]. Recently it has been reported that ACLR using BPTB replicates the fibrous arrangement of healthy ligaments [4,5]. Furthermore, a rectangular tunnel at the anatomical insertion site, or the anatomical rectangular tunnel (ART), recreates the biomechanics of a healthy ACL, yielding good clinical results [6,7]. In BPTB ACLR incorporating the anatomical rectangular tunnel (ART BPTB ACLR) technique, the pattern of force sharing was similar to that in the normal ACL in response to anterior tibial load and during passive knee extension motion [8]. We previously reported a safe, minimally invasive method of ART BPTB ACLR technique by using a rectangular retro-dilator (RRD) (Ario Medical, Osaka, Japan) [9]. The RRD method enables easier bone socket and tunnel creation at the intended target positions whilst minimizing bone drilling. In this study, we evaluated the femoral bone socket and tibial bone tunnel aperture positions made with the RRD, which we manufactured specifically for ART BPTB ACLR.

## Methods

Primary ACLR with BPTB grafts using RRD were performed in 56 patients from April 2015 to August 2017. Bone socket and tunnel aperture positions were evaluated in 42 patients (all male, with a mean age of 29 years, range: 17–51 years), who agreed to undergo a CT evaluation post-operatively. At 3 weeks after surgery, a 3-D CT of the operated knee joint was taken. The centers of the femoral bone socket and the tibial bone tunnel apertures were measured where the diagonal lines of the rectangle crossed and then evaluated using the quadrant method [10,11]. The aperture centers of the femoral bone socket and the tibial bone tunnel were measured using Image J software (National Institute of Health, Bethesda, Maryland) 12] with the average of two independent readings measured by two orthopaedic surgeons used for analysis. Regarding the femoral bone socket, in order to clarify the positional relationship with the resident’s ridge, evaluation was carried out by the method suggested by Magnussen *et al* [13]. In addition, surgical complications were also investigated in all cases. Written informed consent was obtained from all participants prior to surgery. Consent was obtained for both medical treatment and for participation in this study. This reconstruction technique was considered standard of care at our institution, and its outcome was investigated in this study. All protocols in this study are under approval by the ethics committee for clinical research at The Jikei University School of Medicine [Permission no. 30–458 (9479)].

### Surgical procedure

**Fig 1:**
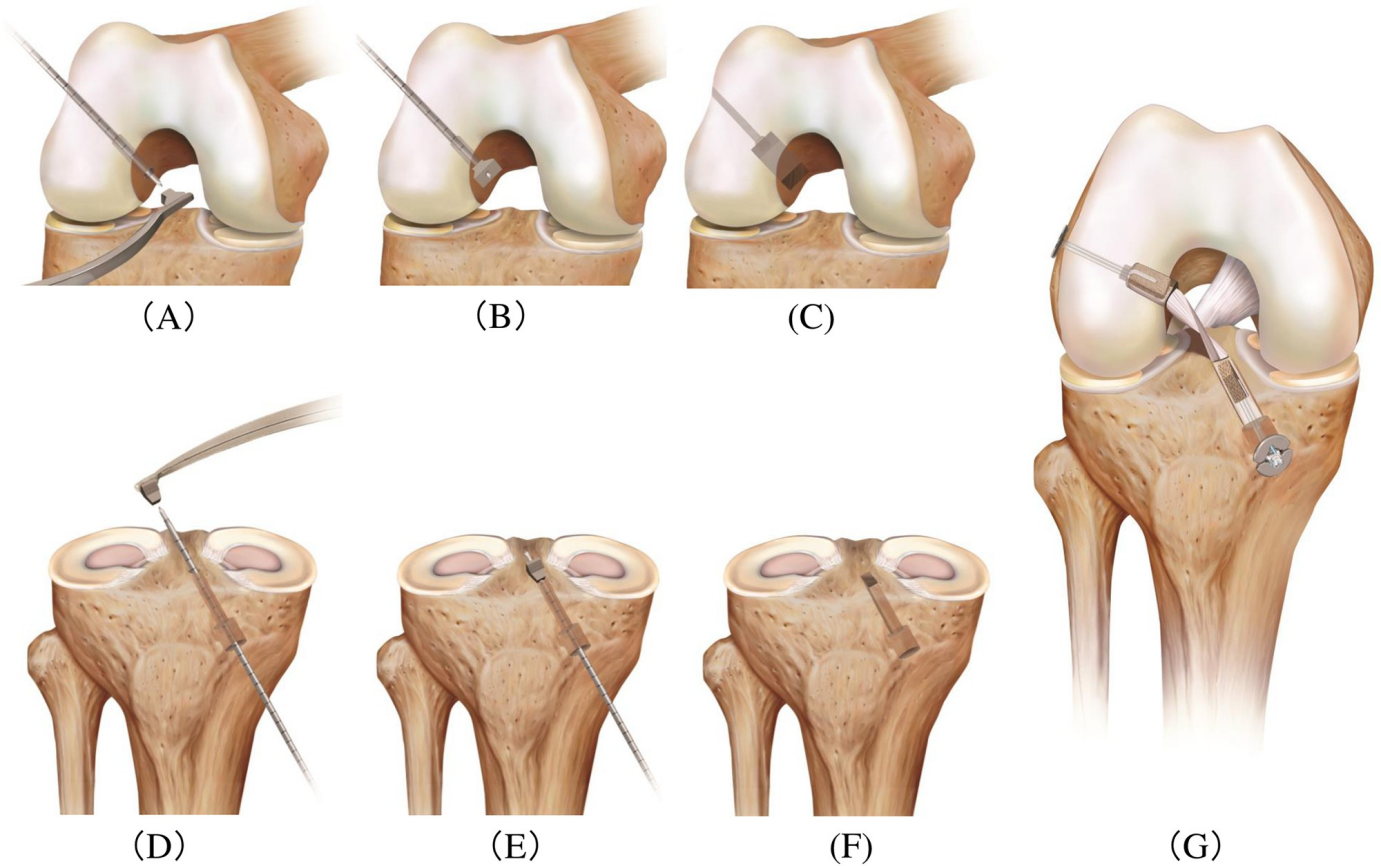
Femoral socket and tibial tunnel preparation of right knee. (A) After a round bone tunnel (6 mm in diameter and 25 mm deep) has been created in a retrograde fashion with the Short FlipCutter II, the dilator (arrow) is set on the 3.0-mm guide pin using the RetroConstruction Drill Guide within the knee joint. (B) The rounded edge of the rectangular retro-dilator (arrow) is inserted into the femoral bone tunnel; the dilator fits tightly into the tunnel. The dilator is then inserted into the femoral bone. (C) A rectangular bone tunnel is created in the femoral bone. (D) The cortical bone is drilled with a 10-mm drill, and then the bone is drilled with a 6.0-mm drill up to the joint space. A rectangular pull-type dilator (arrow) is attached to the retrograde guide, mounting the dilator to the 3.0-mm guide pin within the knee joint. (E) The rectangular retro-dilator (arrow) is inserted into the tibial bone. (F) A rectangular bone tunnel is created in the tibial bone. (G) Completed retro-dilator anatomic rectangular tunnel bone-patellar tendon-bone anterior cruciate ligament reconstruction image. The twist of the ligament can be reproduced.

**Fig 2:**
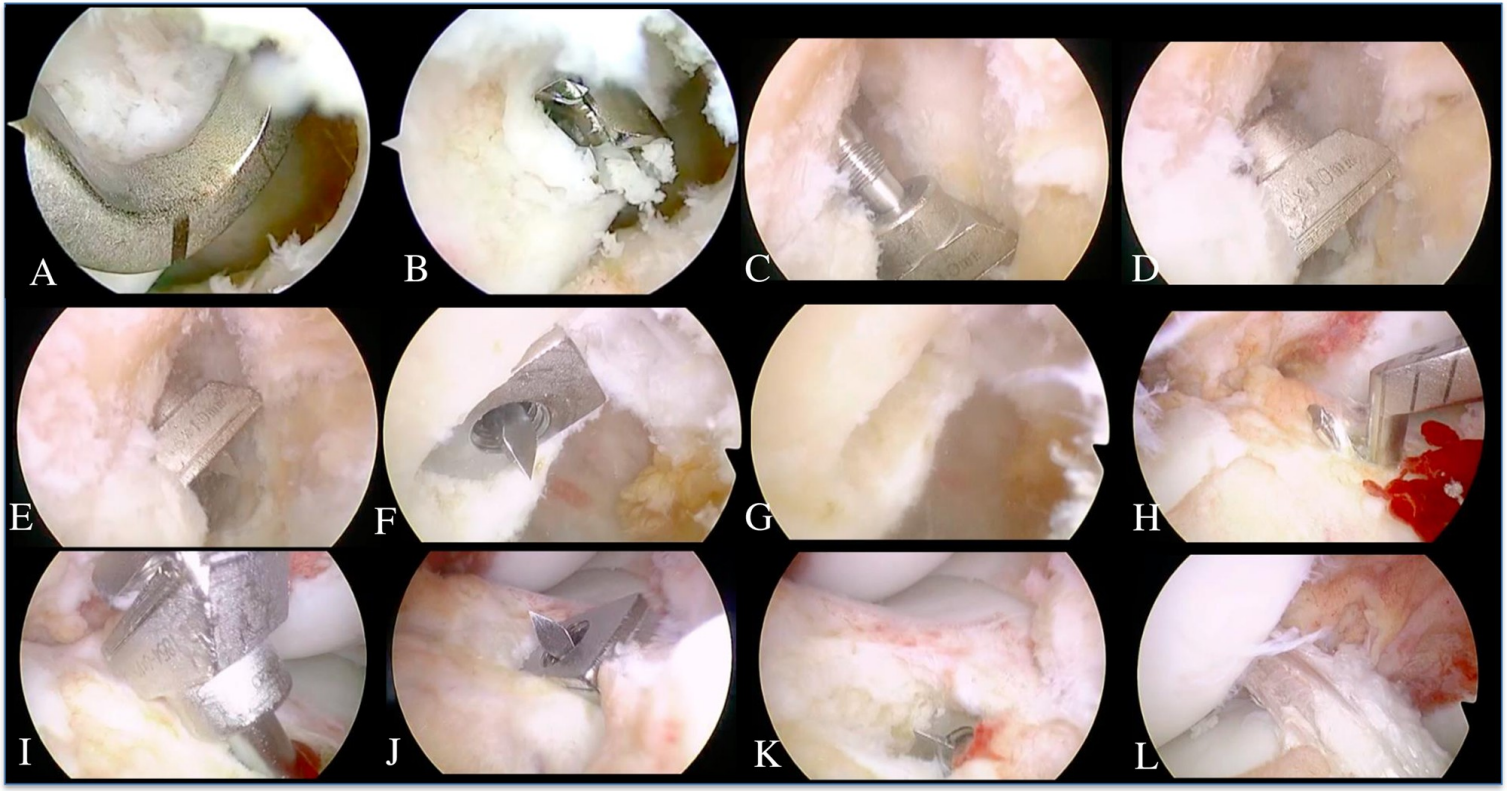
Arthroscopic views of the right knee during surgery. Arthroscopic views of the right knee through anteromedial (A-G) and anterolateral portals (H-L). (A) A 3.5-mm guide pin is inserted with a reference point at the center portion of the anterior cruciate ligament (ACL) femoral attachment behind the resident’s ridge. (B) A round bone tunnel with a diameter of 6.0 mm is created in a retrograde manner with the Short FlipCutter II. (C, D) The dilator is set on the 3-mm RetroDrill Guide Pin using the RetroConstruction Drill Guide within the knee joint. (E) The rounded edge of the rectangular retro-dilator is inserted into the femoral bone tunnel. (F, G) After the dilator’s direction is confirmed, the dilator is pulled into the femoral bone to about 18 mm depth and the rectangular bone socket is created. (H) A guide pin is inserted with the reference guide at the center portion of the ACL tibial attachment. (I) The dilator is set on the 3.0-mm RetroDrill Guide Pin using the RetroConstruction Drill Guide within the knee joint. (J) The rounded edge of the rectangular retro-dilator is inserted into the tibial bone tunnel. (K) After the dilator’s direction is confirmed, the dilator is pulled out of the tibia and the rectangular bone tunnel is created. (L) Arthroscopic view after retro-dilator anatomic rectangular tunnel bone-patellar tendon-bone (BTB) ACL reconstruction.

Surgery was performed as described previously (Figs 1 and 2) [9]. Briefly, surgery was performed in the supine position under general anesthesia, and utilized a 30 ° oblique arthroscope with a diameter of 4.0 mm. The BPTB grafts were harvested by using a two-transverse-incision technique [14] through a 4–5 cm transverse skin incision at the lower border of the patella and through a 2–3 cm transverse skin incision at the level of tibial tuberosity. A parallel knife (Arthrex, Naples, FL) was used to resect the central portion of the patellar tendon. The bone fragment shapes were adjusted with a bone-fragment shaper into rectangular parallelepipeds; each 6 mm thick, 10 mm wide and 13–15 mm long. These fragments were ensured to snugly fit the graft-sizing template before insertion into the bone sockets.

Arthroscopy was performed through medial and lateral infra-patellar portals inserted through the two aforementioned skin incisions. First, a 3.5 mm guide pin was inserted aiming for the center portion of the ACL femoral attachment; posterior to the resident’s ridge and midpoint of antero-medial and postero-lateral bundle attachments. The positions of AM and PL were confirmed by endoscopy, and marked. A guide pin was then inserted aiming at the marked site. Next, a round bone socket (6.0 mm in diameter and 25 mm deep) was created in a retrograde manner using Flip Cutter (Arthrex, Naples, FL). The intra-articular insertion of the dilator was performed through a portal (about 10 mm) slightly larger than the normal portal. Then, the dilator was attached to the 3.0 mm guide pin using RetroDrill System (Arthrex, Naples, FL) within the knee joint and the rounded edge of the RRD was inserted into the femoral bone tunnel. The direction of the dilator was adjusted under fluoroscopy before insertion, ensuring that the dilator was parallel to the line connecting the center of the antero-medial bundle and the center of the postero-lateral bundle. The dilator was then inserted into the femoral bone using a slide hammer to a 15-mm-depth, thus creating a rectangular parallelepiped bone tunnel. For preparation of the tibial tunnel, a 2.4-mm guide pin was inserted into the center of the ACL footprint, aiming slightly medially under a reference guide. Next, the cortical portion of the tibia was drilled in 10 mm with a 10-mm drill, in order to prevent fracture when pulling out the dilator from the tibial bone tunnel.

**Fig 3:**
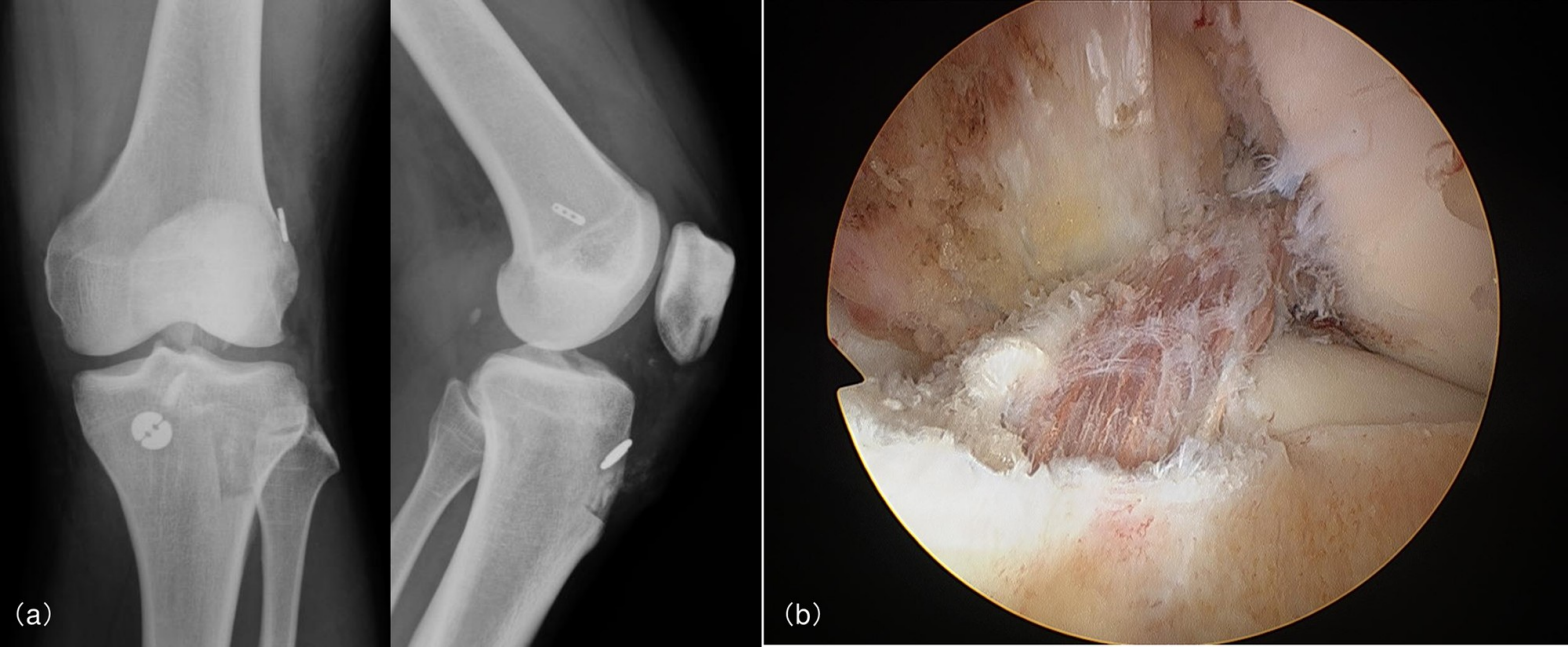
Post-operative radiographs and arthroscopic view of the left knee after surgery. (a): Post-operative radiographs ((fixation to the femur by BTB TightRope (Arthrex, Naples, FL) and to the tibia by ABS button (Arthrex, Naples, FL)) (b): Arthroscopic view.

Subsequently, the tibial bone tunnel was prepared in the same fashion as the femoral socket described above. BPTB graft was fixed to the femoral bone by BPTB TightRope (Arthrex, Naples, FL) and to the tibial bone by ABS button (Arthrex, Naples, FL) (Fig 3).

## Result

Mean patient characteristics were 172.3 cm in height, and 72.1 kg in weight with a BMI of 24.3 kg/m^2^. The mean patellar tendon length measured with the picture archiving and communication system (PACS) measurement tool on the MRI was 47.7 mm (Table 1).

**Table 1:** Pre-operative patient information.

	AVE±S.D.	Range
Gender	Male:42 Female:0	
Age (year)	29.9±10.1	17–51
Height (cm)	172.3±5.0	160.0–181.0
Weight (kg)	72.1±12.9	56.0–115.0
BMI (kg/m^2^)	24.3±4.0	19.0–38.9
Length of patella tendon (mm)	47.7±2.8	44.0–54.8

BMI: body mass index

AVE±S.D.: average±standard deviation

Range: minimum-maximum

**Fig 4:**
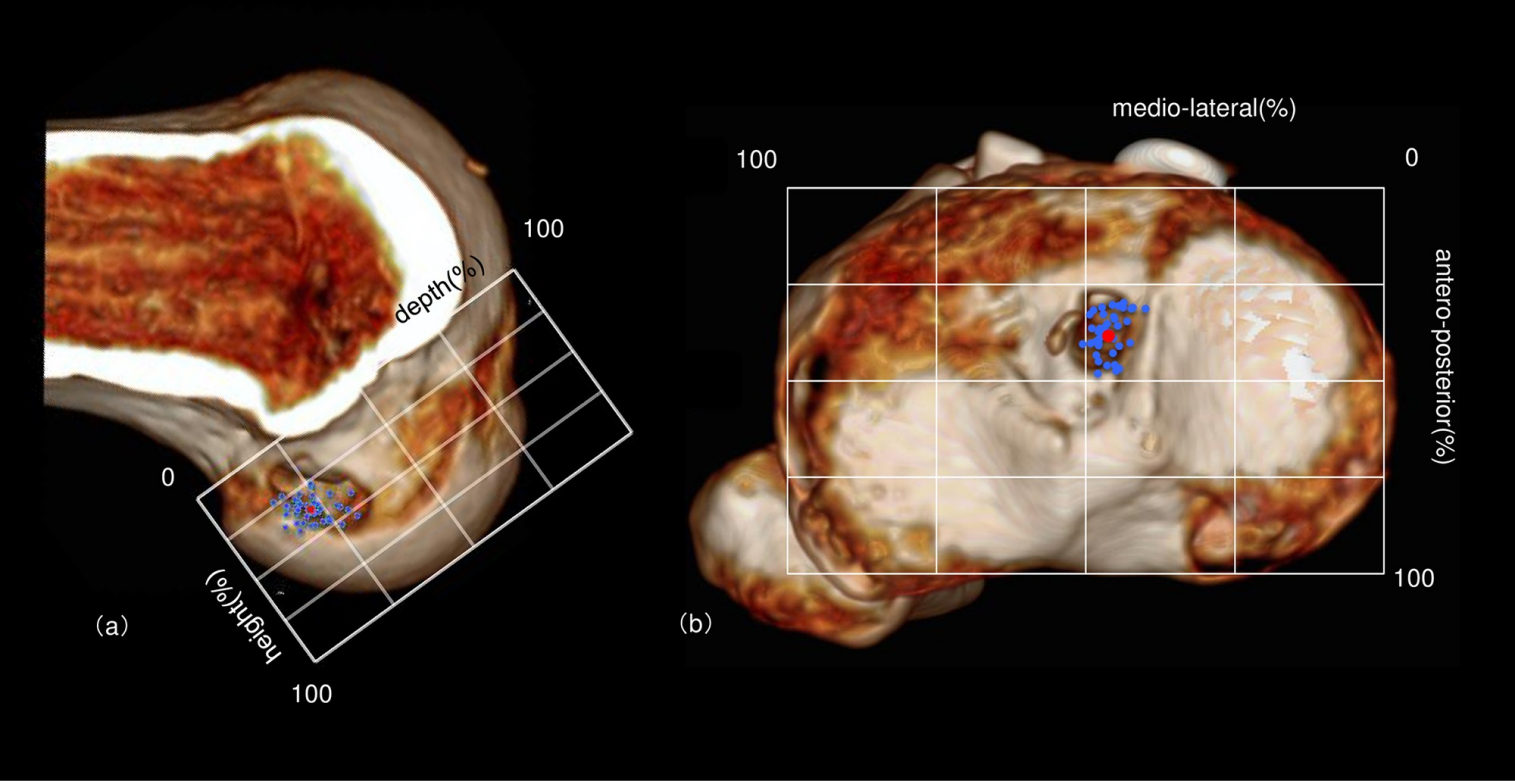
Evaluation of bone aperture positions using the quadrant method on 3-D CT scans. Evaluation of bone aperture positions using the quadrant method on 3-D CT scans taken 3 weeks after the operation. The analysis showed that all bone apertures were placed in anatomically appropriate positions. (a): femur, (b): tibia.

The measurement was carried out on a cross sectional image that passes through the patellar spine on the sagittal image. At 3 weeks after the surgery, the positions of the aperture center of the femoral bone socket and the tibial bone tunnel were evaluated by 3-D CT using the quadrant method [9,10]. In our series, the depth of aperture center of the femoral socket was 22.0 ± 4.2% along the Blumensaat’s line and the height was 37.4 ± 7.2% from the Blumensaat’s line (Fig 4a). The aperture center of the tibial tunnel was located 37.7 ± 5.2% antero-posteriorly, and 46.1 ± 2.2% medio-laterally (Fig 4b).

In addition to this, the apertures were assessed using the Magnussen classification [13], which categorized the positional relationship between the femoral socket aperture and the resident’s ridge, into three groups. 39 cases (92.9%) were classified as type 1, where the aperture is located behind the resident’s ridge. 3 cases (7.1%) were classified as type 2, which overlapped with the resident’s ridge. None of the cases were classified as type 3, which is deemed as not anatomical. The results of 3-D CT evaluation demonstrated that almost all femoral sockets were made in the anatomically appropriate position.

In all cases, intra-operative complication rates were evaluated. Severe complications such as damage to nerves or blood vessels did not occur intra-operatively. However, in 2 patients, the bone fragments on the femoral side were accidently damaged during insertion at a relatively early stage of the procedure. The bone fragment had fractured from the insertion site of BPTB Tight Rope. The split extended away from the tendon, but the bone-tendon interface remained intact. In retrospect, the most likely cause for this was excessive force used to pull the bone fragment. The problem was overcome by turning the BPTB upside down, and a fiber TAG was sewn to the tendon segment adjacent to the fracture bone plug for femoral insertion. BPTB Tight Rope was attached to the bone fragment without the fracture for use in the tibial tunnel. As a result, no resulting clinical problems ensued. In addition to this, there were some patients with a long patellar tendon (Table 1). In cases where such graft-tendon length mismatch were likely to occur, the angle of insertion of the guide pin relative to the articular surface was increased so that the tibial bone tunnel became longer. Usually, the femoral guide was set at 105 degrees, and a guide pin was inserted at approximately 45 degrees from the posterior condyles of the femur. The tibial guide is set at 55 degrees, and a guide pin is inserted slightly towards the AMB attachment of the ACL at the tibia. The length of the tibial tunnel can be increased by increasing the tibial guide to 60 or 65 degrees. If graft-tendon mismatch still remained, the tibial bone plug was shortened before fixation. Application of these techniques overcame any problems with regards to tendon length.

## Discussion

Recently, good clinical results of ART BPTB ACLR have been reported [4–7]. Rectangular femoral ACL fixation constructs and grafts may prove more efficacious at restoring in vivo ACL kinematics than round femoral tunnels [7]. Some studies [3,15,16] indicated that bone tunnels can be safely and accurately created with an outside-in technique using retrograde drills, namely: AI drill (Aimedic MMT (Telos Japan), Tokyo, Japan) 17], O-drill (Meira Co., Aichi, Japan), Endobutton retro drill (Smith&Nephew, Andover, MA) [18] and FlipCutter (Arthrex, Naples, FL) [17–20]. We reported a technique of minimally invasive ART BTB ACLR, which utilized the outside-in method and in which a rectangular pull-type dilator (rectangular retro-dilator) was used [9]. In this study, a guide pin was inserted into the anatomical position taking into account the shape of the RRD, and it was confirmed that rectangular socket and tunnel were created at the anatomical position with high probability without complications of posterior wall damage. The 3-D CT evaluation of BPTB ACLR using the RDD confirmed that both the femoral socket and the tibial tunnel were placed in their appropriate anatomical positions as shown in similar studies reported previously [21–29] (Table 2).

**Table 2:** Comparison between the cadaver study results using the quadrant method.

author	year	n	femoral socket (%)		tibial tunnel (%)		study
			depth*	height**	antero-posterior	medio-lateral	
Colombet et al.[21]	2006	7	29.4	36.5	-	-	Cadaveric & Radiologic(Xp)
Zantop et al.[22]	2008	20	23.9	38.0	-	-	Cadaveric & Radiologic(Xp)
Tsukada et al.[23]	2008	36	30.4	30.0	43.9	48.9	Cadaveric & Photographs
Lorenz et al.[24]	2009	12	24.0	33.5	46.5	51	Cadaveric & Radiologic(CT)
Guo et al.[25]	2009	16	43.1	38.3	-	-	Cadaveric & Radiologic(Xp)
Iriuchishima et al.[26]	2010	15	23.5	39.0	40.5	48	Cadaveric & Radiologic(Xp)
Forsythe et al.[27]	2010	8	28.4	44.3	35.7	51.5	Cadaveric & Radiologic(CT)
Pietrini et al.[28]	2011	12	25.3	28.5	43.7	47.2	Cadaveric & Radiologic(Xp)
Lee et al.[29]	2015	15	36.5	39.7	39.5	50.6	Cadaveric & Radiologic(CT)
mean			29.4	36.4	41.6	49.5	
This study			22.0	37.4	37.7	46.1	

The anatomical positions of femoral insertion site and tibial tunnel were compared between previous literatures with current study results.

depth*(%) = a/t t: the total sagittal diameter of the lateral femoral condyle along Blumensaat’s line. a: the distance of the tunnel center from the deepest subchondral contour.

height**(%) = b/h h: the maximum intercondylar notch height. b: the distance of the tunnel center from Blumensaat’s line.

**Fig 5:**
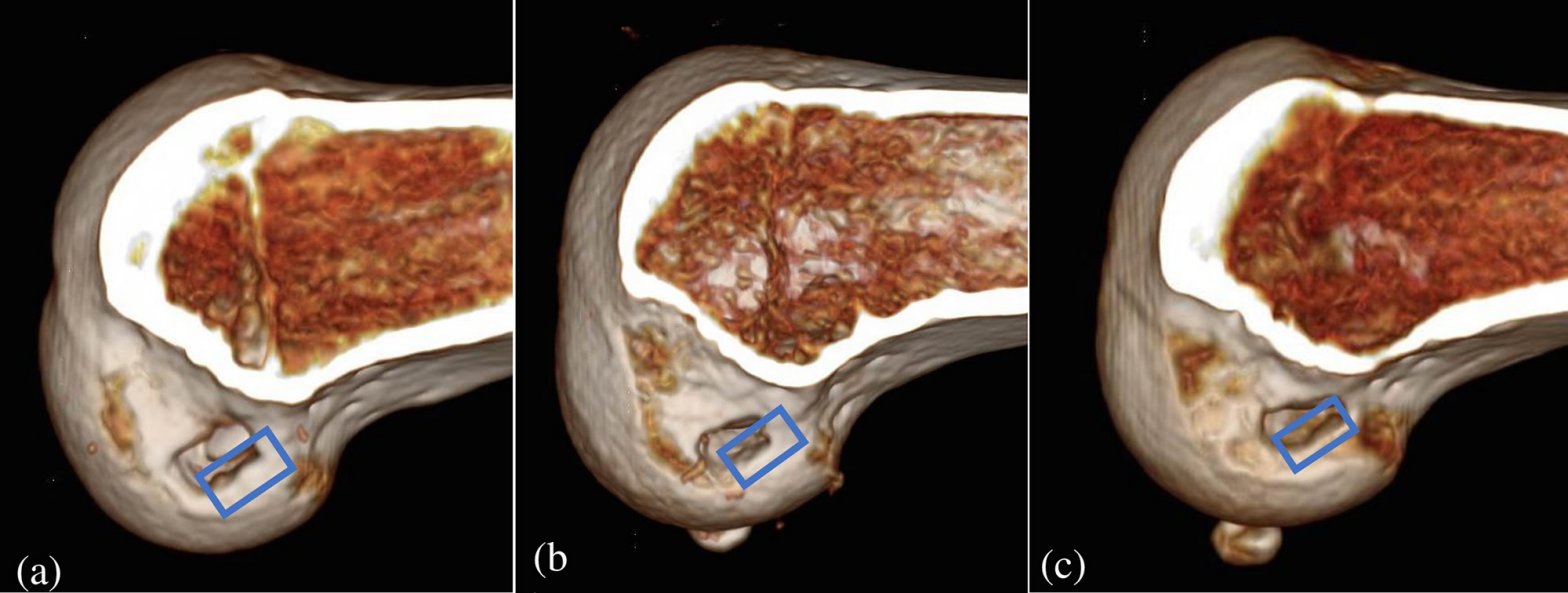
3 cases of Magnussen classification type2. Two cases (a, b) were considered technical errors at the time of insertion of the guide pin, and one (c) was due to the incorrect orientation of the RRD. An outline of the ideal tunnel arrangement has been shown in Fig 5 (blue square).

In addition to this, the Magnussen classification [13], which classified the positional relationship between the femoral socket aperture and the resident’s ridge into three groups, showed that almost all femoral sockets were anatomically created. Out of 42 cases, 3 cases were classified as type 2 but they were anteriorly positioned, showing only a small overlap with the resident’s ridge (Fig 5). Two cases were considered technical errors that occurred upon insertion of the guide pin (Fig 5a and 5b), and one was due to incorrect orientation of the RRD (Fig 5c). In a previous study of round tunnel BTB ACLR performed by the outside-in method, 104 cases out of 135 cases were Type 1 (77.0%), 28 cases were Type 2 (20.8%), and 3 cases were Type 3 (2.2%), demonstrating a relatively high anatomical rate. In our study, a high ratio of Type 1 was achieved because the bone aperture using the RRD is rectangular and its shape allows for a better fit at the ACL footprint compared to a large round tunnel. In the 3 Type 2 cases in this study, the bone aperture center was on or behind the resident’s ridge, and furthermore no severe intra-operative complications occurred in our series such as posterior wall penetration, articular cartilage damage, or neurovascular injury.

Our procedure has the following advantages: 1) the femoral bone socket can be created at the target position more safely and easily than with the trans-tibial or the antero-medial portal techniques [30,31]; 2) the amount of harvested bone necessary for graft preparation can be reduced to a minimum when compared to the conventional outside-in technique; 3) the risk of technical failure is relatively low since the guide pin is inserted only once; 4) time required for the operation can be reduced due to procedural simplicity, compared to the original rectangular BPTB surgery that requires two guide pins using the outside-in method. On the other hand, pitfalls of the procedure include: 1) in cases where the penetration length of the guide pin of the femur is short, the distance between the bone socket and the fixture is too close, resulting in increased risk of bone tunnel penetration and fracture; 2) possibility of damage to the bone tunnel and its surrounding structures due to excess intra-operative force applied; 3) difficulty of dilator positioning in cases of ACL damage with a preserved remnant or in those with a narrow intercondylar fossa; 4) possibility of fracture in cases with pathologically weakened bones such as in patients with osteoporosis.

Although our proposed ART BPTB ACLR carries a few risks of intra-operative complications, it is a simpler and less invasive method of ART BPTB ACLR, provided that indications for the technique are carefully considered and that the operative techniques are appropriately applied. A major pitfall of this technique, is the difficulty in inserting the BPTB graft, due to its rectangular shape. Possible complication is the fracture of the bone fragment, which can be prevented by rounding the tip of the bone fragment and carefully guiding it into the socket with a probe. Also, one downside of this rectangular technique is that graft tunnel mismatch can occur. If the length of the graft is too long when measuring the BPTB graft, the graft length can be adjusted by shortening the bone fragment on the tibial side. Also, by increasing the tibial angle when making the tibial bone tunnel, length mismatch can be prevented. If mismatch still remains, the tibial bone can be removed to fix the Fiber TAG (Arthrex, Naples, FL) directly to the patellar tendon, but in the Japanese population, the patellar tendon is often about 40mm in length and there is almost no mismatch.

33 cases out of 42 cases were observed for over 1 year. Return to competitive level prior to injury was achieved in 78.8% with an average return time of 10.4 ± 2.5 months. There were 4 incidences of recurrence; all were due to poor compliance, and returned to sports activities without undergoing performance tests such as the hop test, which is our criteria for return to sports. 3 cases were recurrence within 1 year of surgery. Overall, 22 cases (66.7%) returned to sports without recurrence, which is comparable to the reported return rate of 65% to sports pre-injury [32]. In the future, it is necessary to follow up on the long-term outcome and examine the clinical results after surgery.

## Conclusion

BPTB ACLR using the RRD is a safe technique that enables bone socket and tunnel creation at the anatomical positions.

## Supporting information

S1 Table Patient information. Age, height, weight, BMI and patella tendon length of the operated patients.
(XLSX)

S2 Table Measurement of bone tunnel positions. Positions of bone tunnels independently measured by two Orthopaedic surgeons.
(XLSX)

S1 Dataset 3DCT image of femoral and tibial tunnels for all cases. Data required to calculate and replicate all the figures and tables.
(ZIP)
