# Supplementary figures and images for: Positioning the femoral bone socket and the tibial bone tunnel using a rectangular retro-dilator in anterior cruciate ligament reconstruction
Source: PLoS One. 2019 May 2;14(5):e0215778. doi: 10.1371/journal.pone.0215778 (PMC6497238; doi:10.1371/journal.pone.0215778)

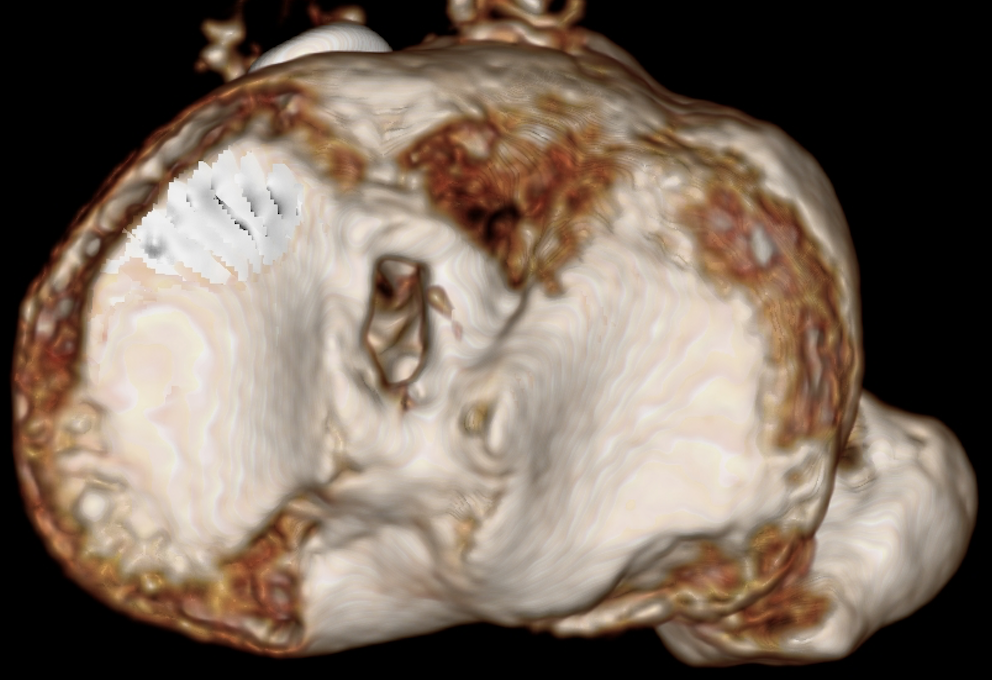

Supplement: S1 Dataset — Data required to calculate and replicate all the figures and tables. (ZIP) [file pone.0215778.s003.zip › 3DCT PLOS ONE/32/tibia.png]

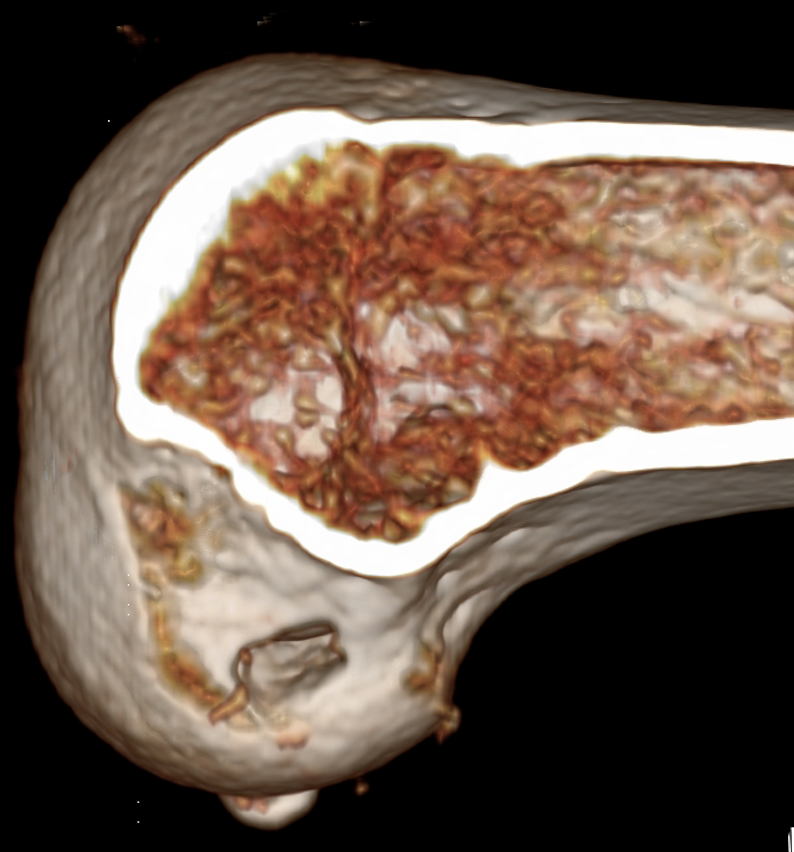

Supplement: S1 Dataset — Data required to calculate and replicate all the figures and tables. (ZIP) [file pone.0215778.s003.zip › 3DCT PLOS ONE/32/femur.png]

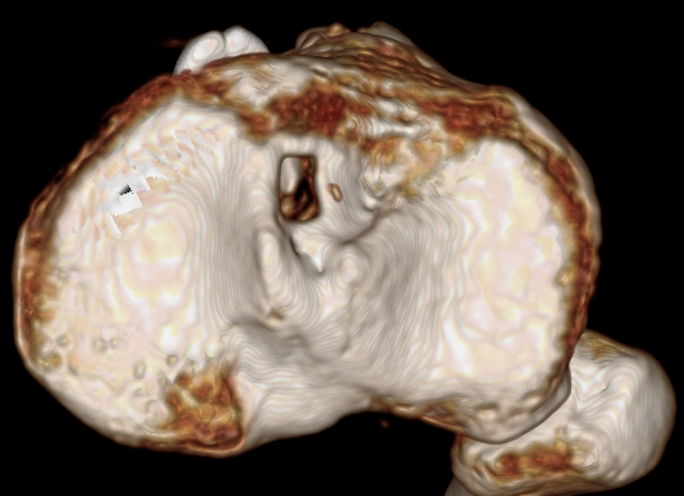

Supplement: S1 Dataset — Data required to calculate and replicate all the figures and tables. (ZIP) [file pone.0215778.s003.zip › 3DCT PLOS ONE/35/tibia.png]

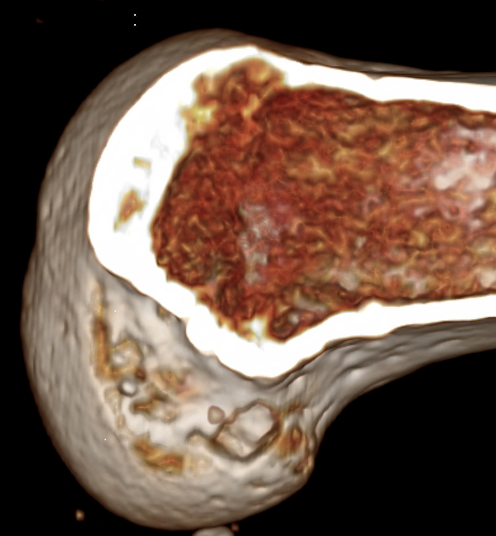

Supplement: S1 Dataset — Data required to calculate and replicate all the figures and tables. (ZIP) [file pone.0215778.s003.zip › 3DCT PLOS ONE/35/femur.png]

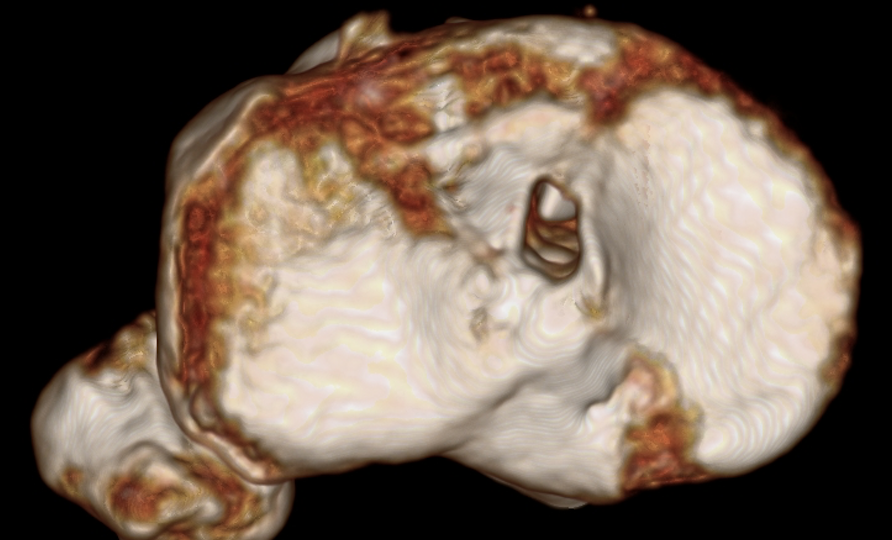

Supplement: S1 Dataset — Data required to calculate and replicate all the figures and tables. (ZIP) [file pone.0215778.s003.zip › 3DCT PLOS ONE/34/tibia.png]

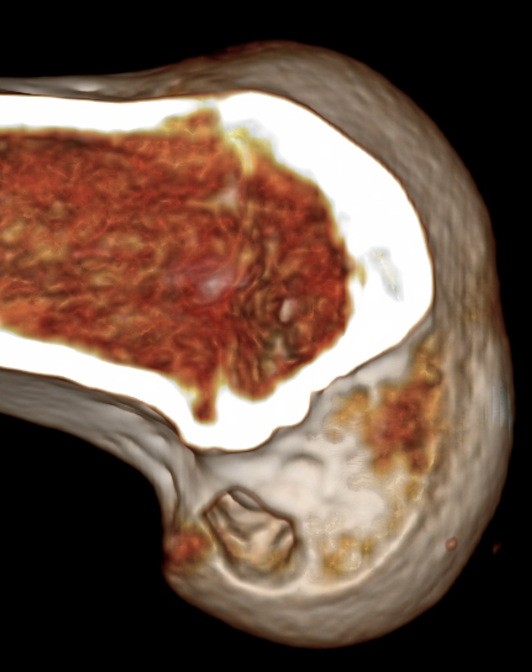

Supplement: S1 Dataset — Data required to calculate and replicate all the figures and tables. (ZIP) [file pone.0215778.s003.zip › 3DCT PLOS ONE/34/femur.png]

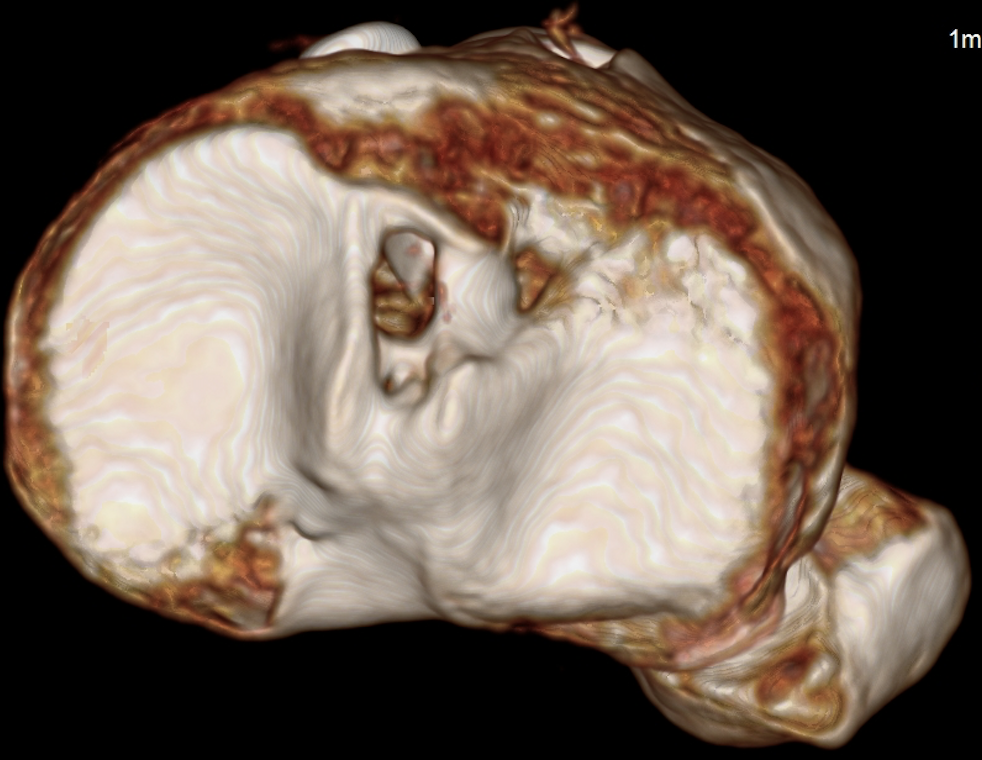

Supplement: S1 Dataset — Data required to calculate and replicate all the figures and tables. (ZIP) [file pone.0215778.s003.zip › 3DCT PLOS ONE/33/tibia.png]

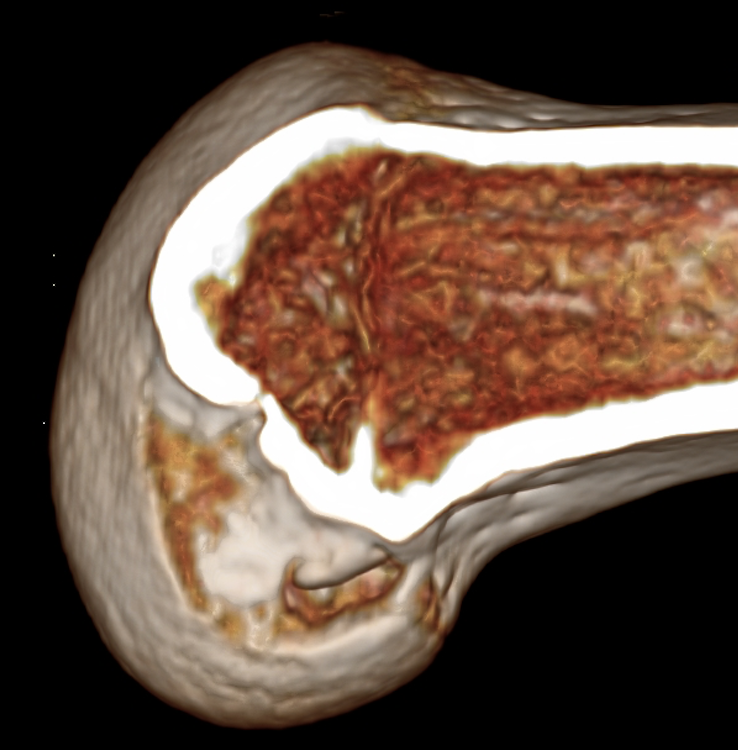

Supplement: S1 Dataset — Data required to calculate and replicate all the figures and tables. (ZIP) [file pone.0215778.s003.zip › 3DCT PLOS ONE/33/femur.png]

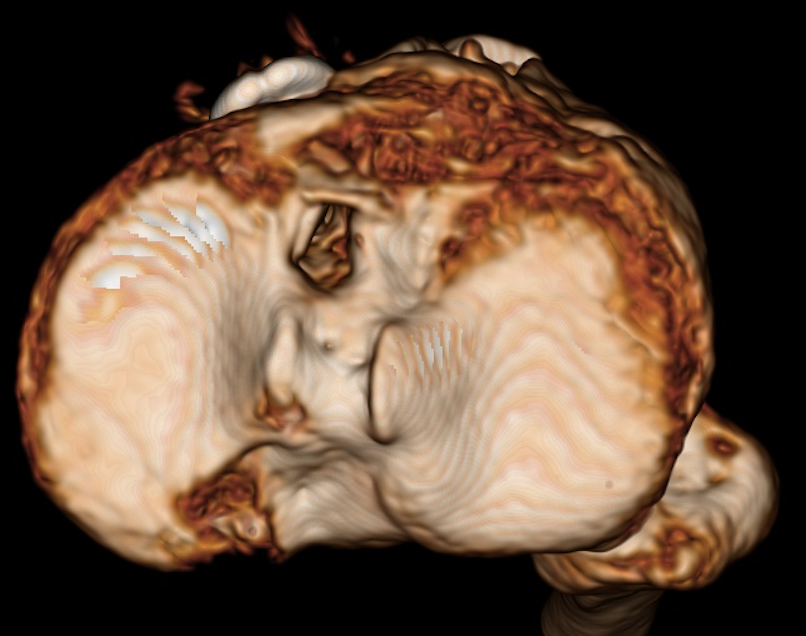

Supplement: S1 Dataset — Data required to calculate and replicate all the figures and tables. (ZIP) [file pone.0215778.s003.zip › 3DCT PLOS ONE/20/tibia.png]

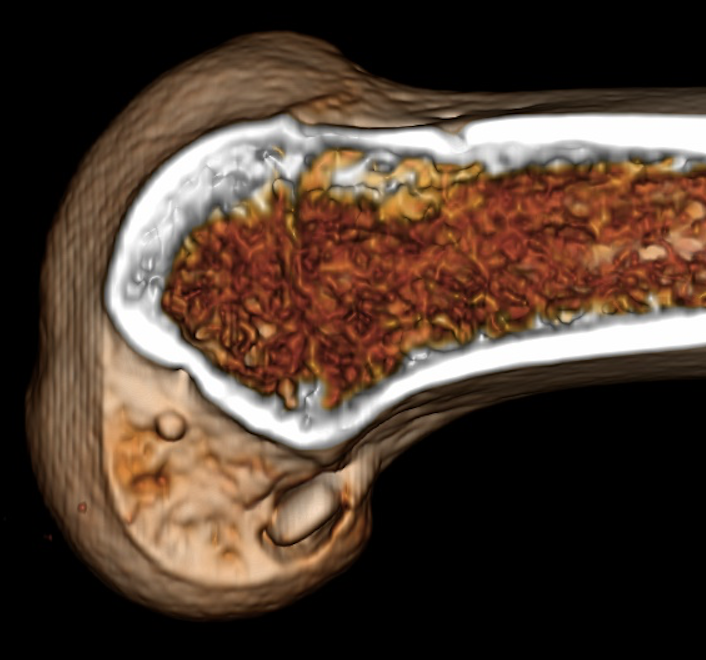

Supplement: S1 Dataset — Data required to calculate and replicate all the figures and tables. (ZIP) [file pone.0215778.s003.zip › 3DCT PLOS ONE/20/femur.png]

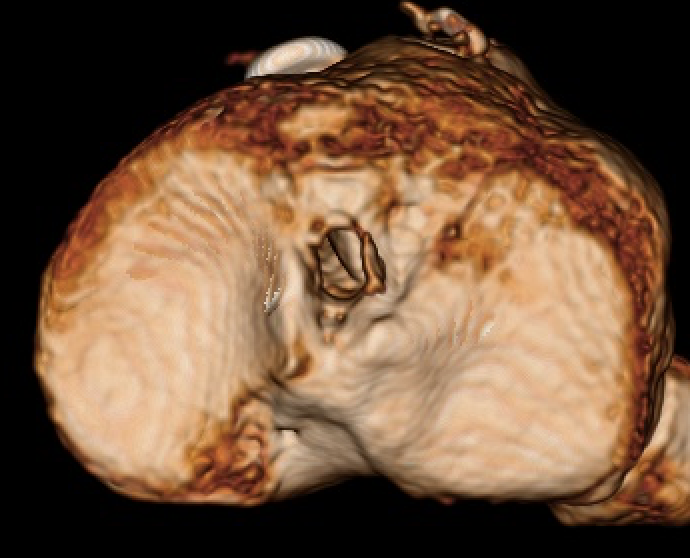

Supplement: S1 Dataset — Data required to calculate and replicate all the figures and tables. (ZIP) [file pone.0215778.s003.zip › 3DCT PLOS ONE/18/tibia.png]

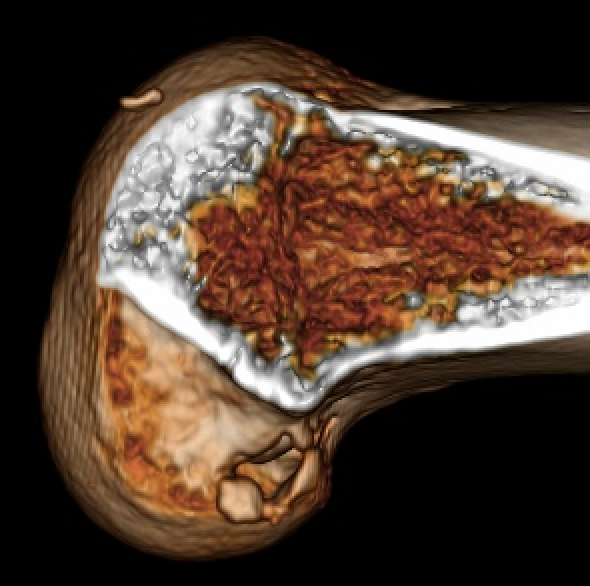

Supplement: S1 Dataset — Data required to calculate and replicate all the figures and tables. (ZIP) [file pone.0215778.s003.zip › 3DCT PLOS ONE/18/femur.png]

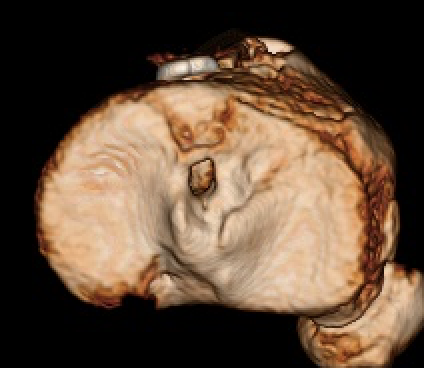

Supplement: S1 Dataset — Data required to calculate and replicate all the figures and tables. (ZIP) [file pone.0215778.s003.zip › 3DCT PLOS ONE/27/tibia.png]

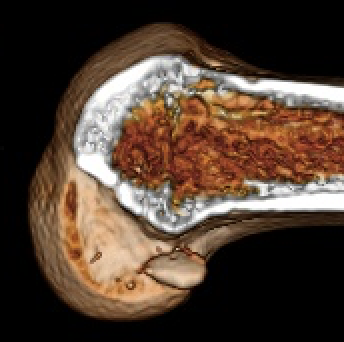

Supplement: S1 Dataset — Data required to calculate and replicate all the figures and tables. (ZIP) [file pone.0215778.s003.zip › 3DCT PLOS ONE/27/femur.png]

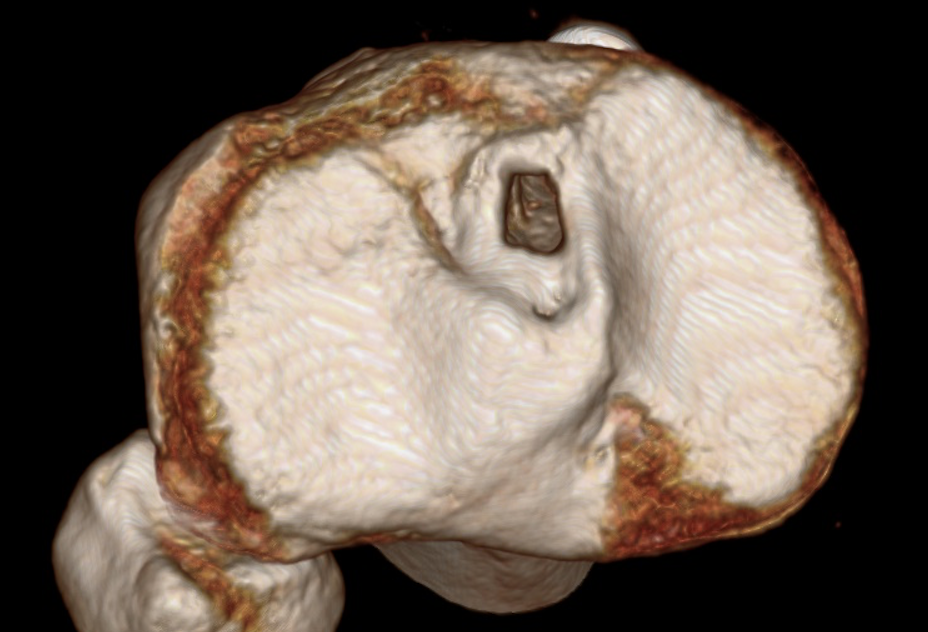

Supplement: S1 Dataset — Data required to calculate and replicate all the figures and tables. (ZIP) [file pone.0215778.s003.zip › 3DCT PLOS ONE/9/tibia.png]

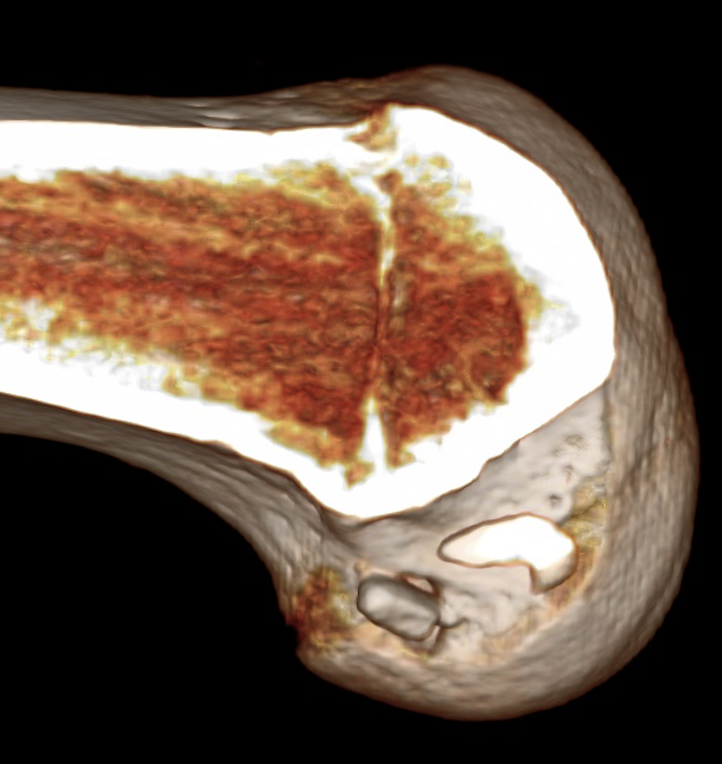

Supplement: S1 Dataset — Data required to calculate and replicate all the figures and tables. (ZIP) [file pone.0215778.s003.zip › 3DCT PLOS ONE/9/femur.png]

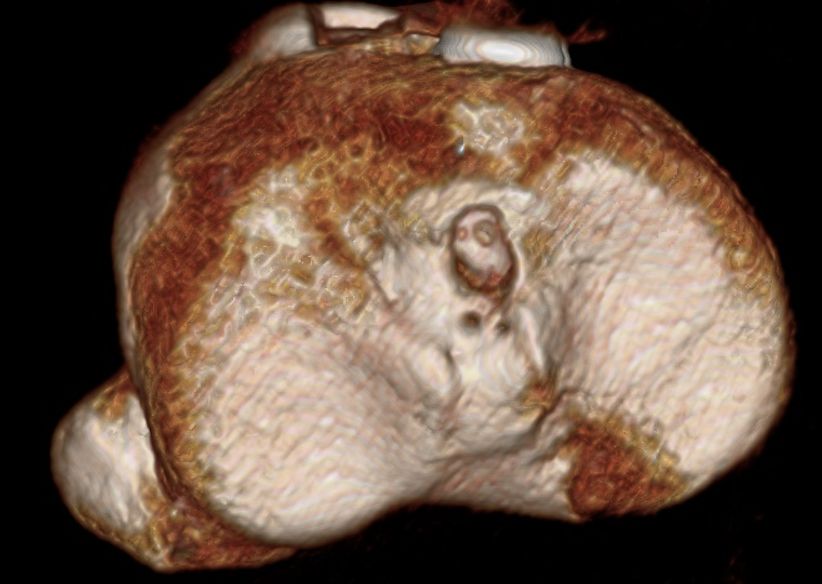

Supplement: S1 Dataset — Data required to calculate and replicate all the figures and tables. (ZIP) [file pone.0215778.s003.zip › 3DCT PLOS ONE/11/tibia.png]

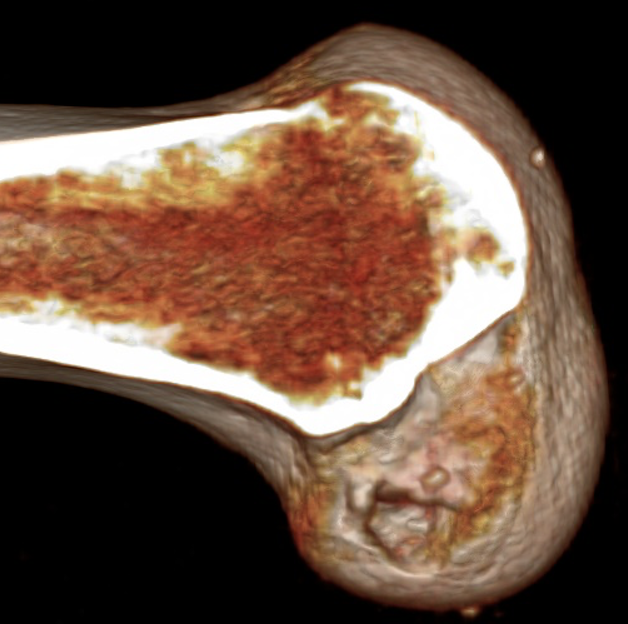

Supplement: S1 Dataset — Data required to calculate and replicate all the figures and tables. (ZIP) [file pone.0215778.s003.zip › 3DCT PLOS ONE/11/femur.png]

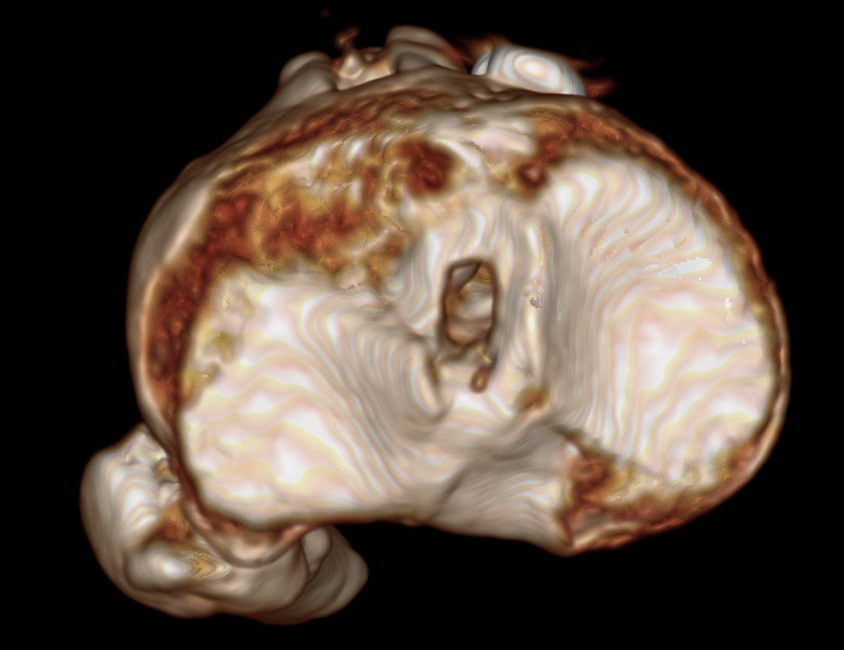

Supplement: S1 Dataset — Data required to calculate and replicate all the figures and tables. (ZIP) [file pone.0215778.s003.zip › 3DCT PLOS ONE/7/tibia.png]

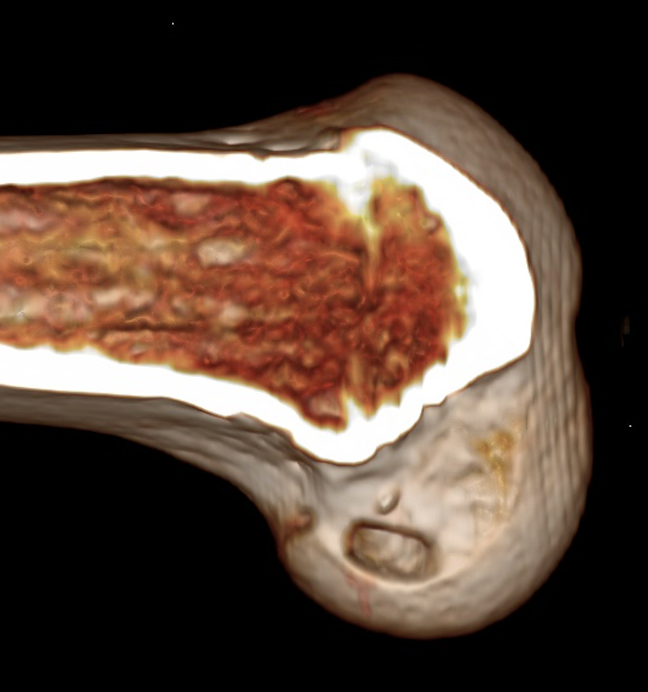

Supplement: S1 Dataset — Data required to calculate and replicate all the figures and tables. (ZIP) [file pone.0215778.s003.zip › 3DCT PLOS ONE/7/femur.png]

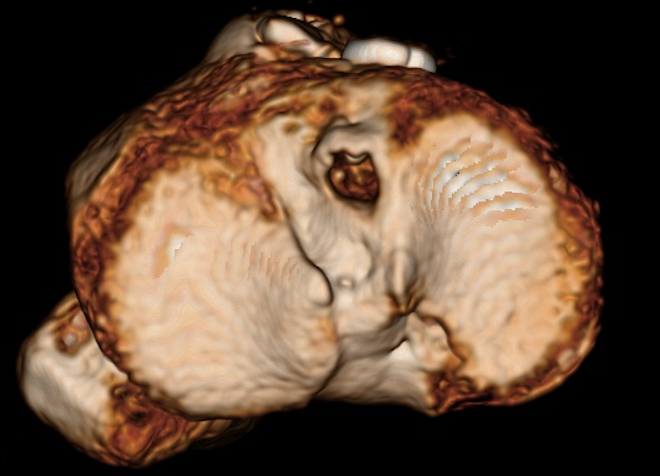

Supplement: S1 Dataset — Data required to calculate and replicate all the figures and tables. (ZIP) [file pone.0215778.s003.zip › 3DCT PLOS ONE/29/tibia.png]

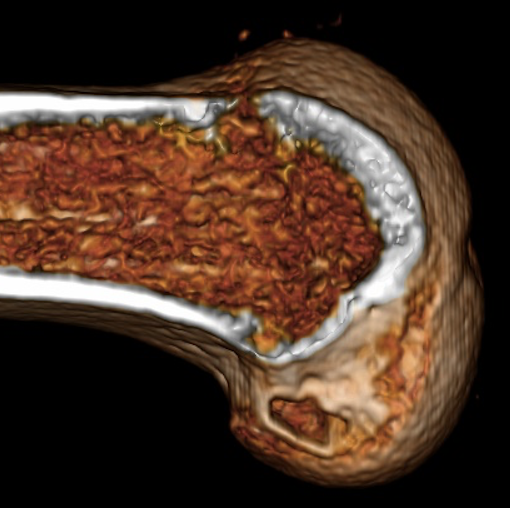

Supplement: S1 Dataset — Data required to calculate and replicate all the figures and tables. (ZIP) [file pone.0215778.s003.zip › 3DCT PLOS ONE/29/femur.png]

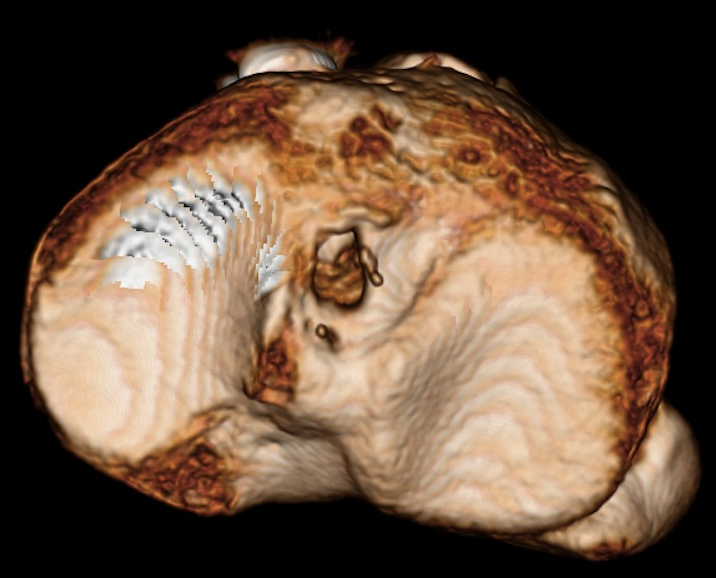

Supplement: S1 Dataset — Data required to calculate and replicate all the figures and tables. (ZIP) [file pone.0215778.s003.zip › 3DCT PLOS ONE/16/tibia.png]

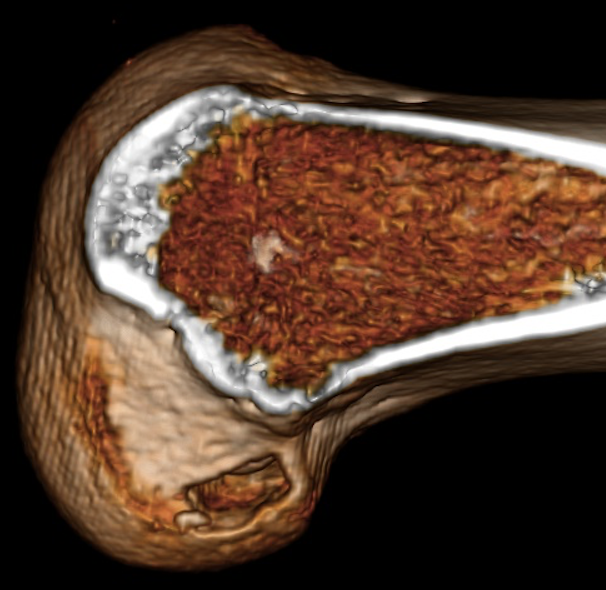

Supplement: S1 Dataset — Data required to calculate and replicate all the figures and tables. (ZIP) [file pone.0215778.s003.zip › 3DCT PLOS ONE/16/femur.png]

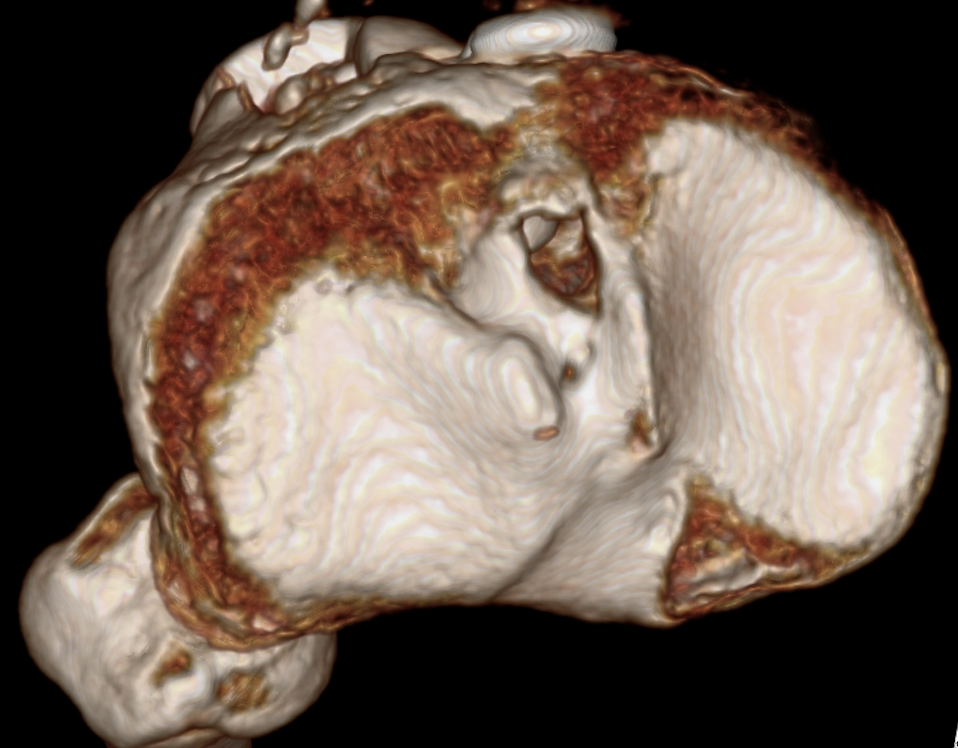

Supplement: S1 Dataset — Data required to calculate and replicate all the figures and tables. (ZIP) [file pone.0215778.s003.zip › 3DCT PLOS ONE/42/tibia.png]

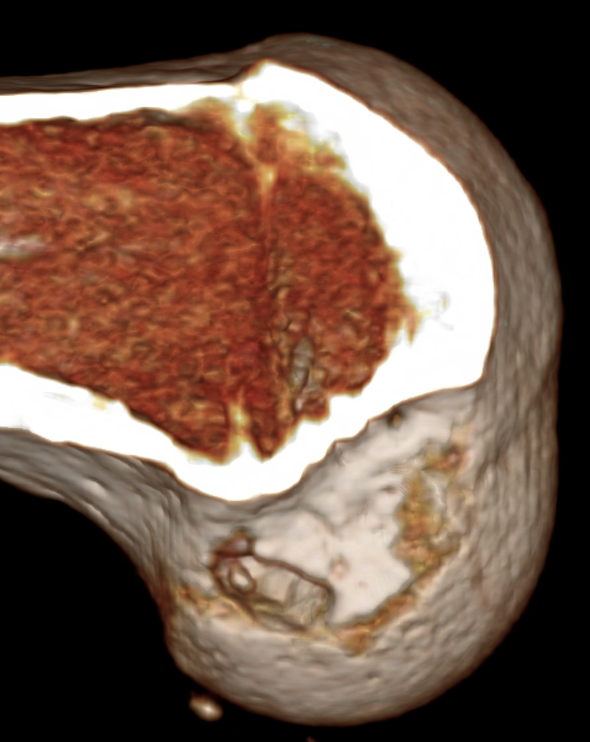

Supplement: S1 Dataset — Data required to calculate and replicate all the figures and tables. (ZIP) [file pone.0215778.s003.zip › 3DCT PLOS ONE/42/femur.png]

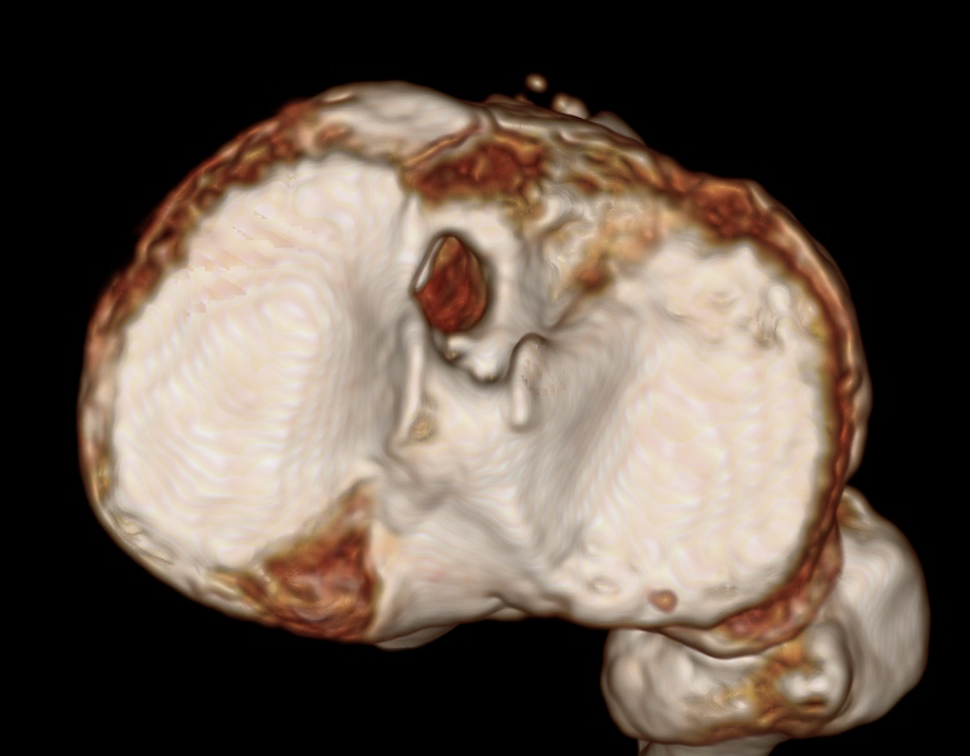

Supplement: S1 Dataset — Data required to calculate and replicate all the figures and tables. (ZIP) [file pone.0215778.s003.zip › 3DCT PLOS ONE/6/tibia.png]

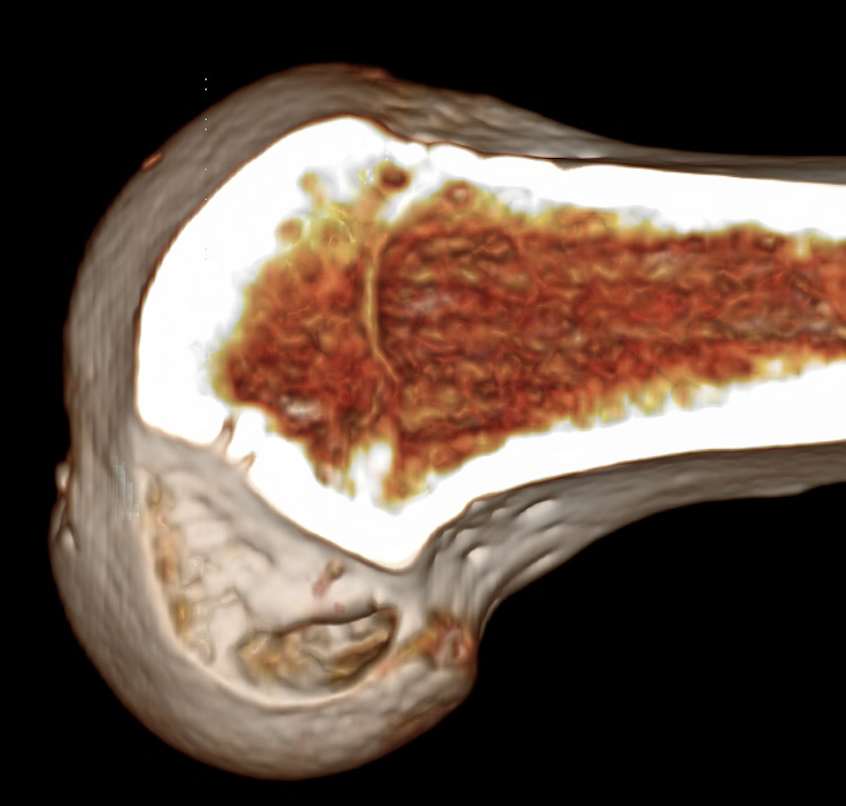

Supplement: S1 Dataset — Data required to calculate and replicate all the figures and tables. (ZIP) [file pone.0215778.s003.zip › 3DCT PLOS ONE/6/femur.png]

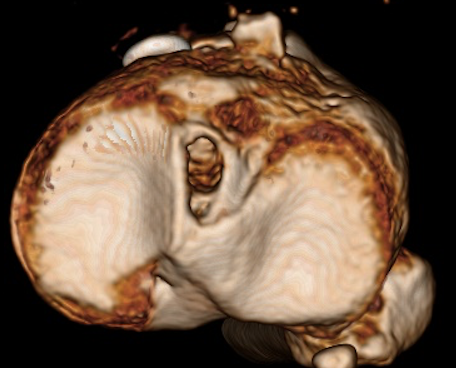

Supplement: S1 Dataset — Data required to calculate and replicate all the figures and tables. (ZIP) [file pone.0215778.s003.zip › 3DCT PLOS ONE/28/tibia.png]

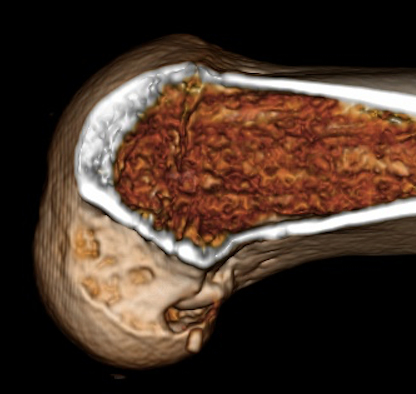

Supplement: S1 Dataset — Data required to calculate and replicate all the figures and tables. (ZIP) [file pone.0215778.s003.zip › 3DCT PLOS ONE/28/femur.png]

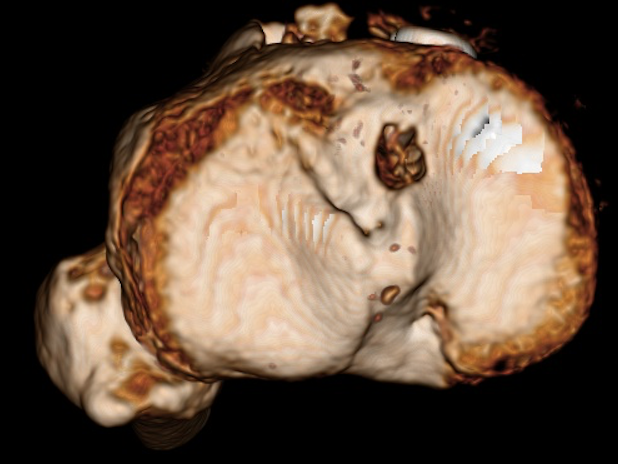

Supplement: S1 Dataset — Data required to calculate and replicate all the figures and tables. (ZIP) [file pone.0215778.s003.zip › 3DCT PLOS ONE/17/tibia.png]

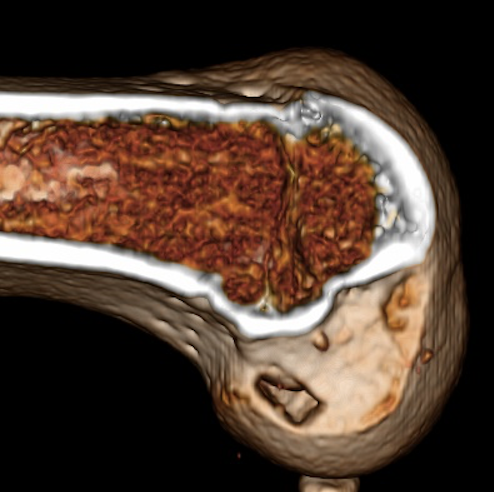

Supplement: S1 Dataset — Data required to calculate and replicate all the figures and tables. (ZIP) [file pone.0215778.s003.zip › 3DCT PLOS ONE/17/femur.png]

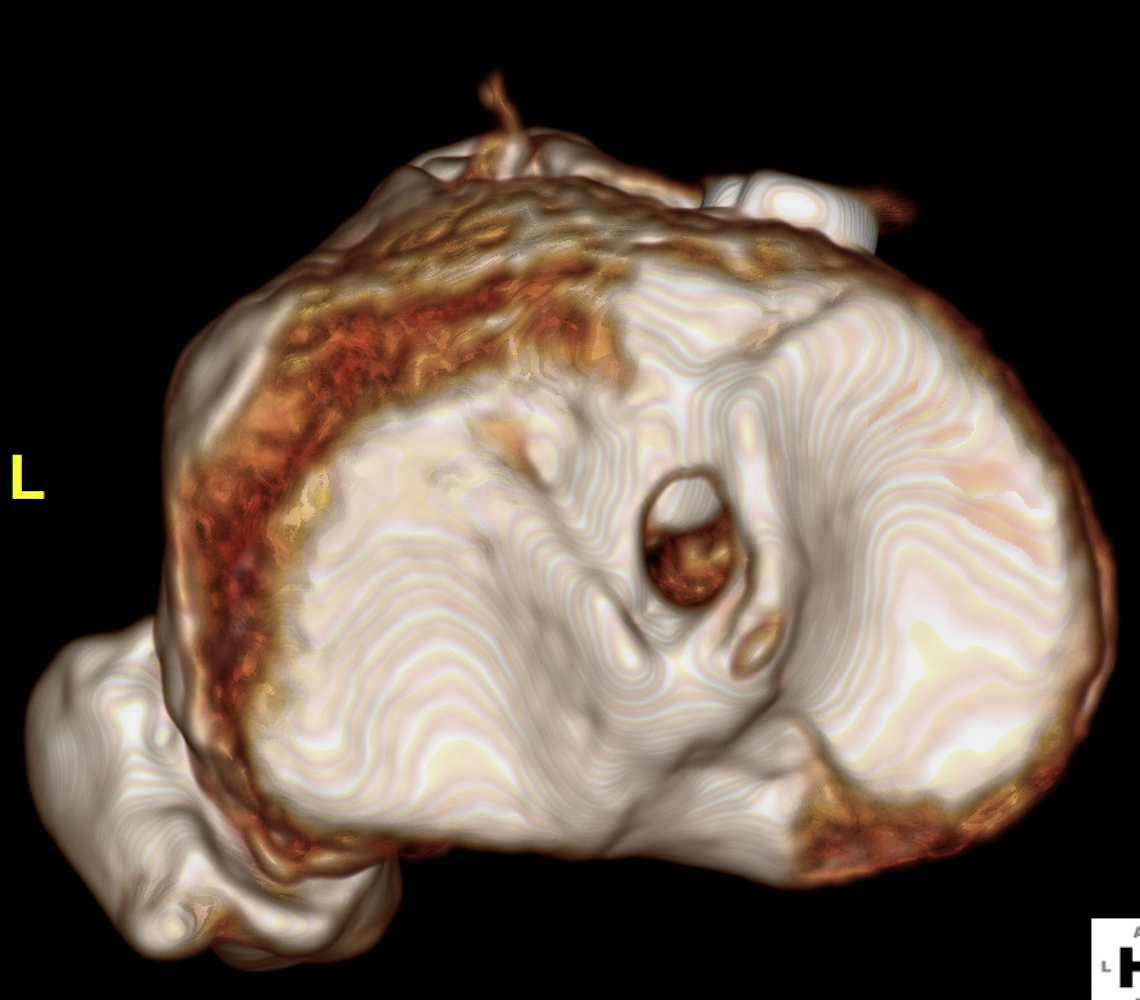

Supplement: S1 Dataset — Data required to calculate and replicate all the figures and tables. (ZIP) [file pone.0215778.s003.zip › 3DCT PLOS ONE/1/tibia.png]

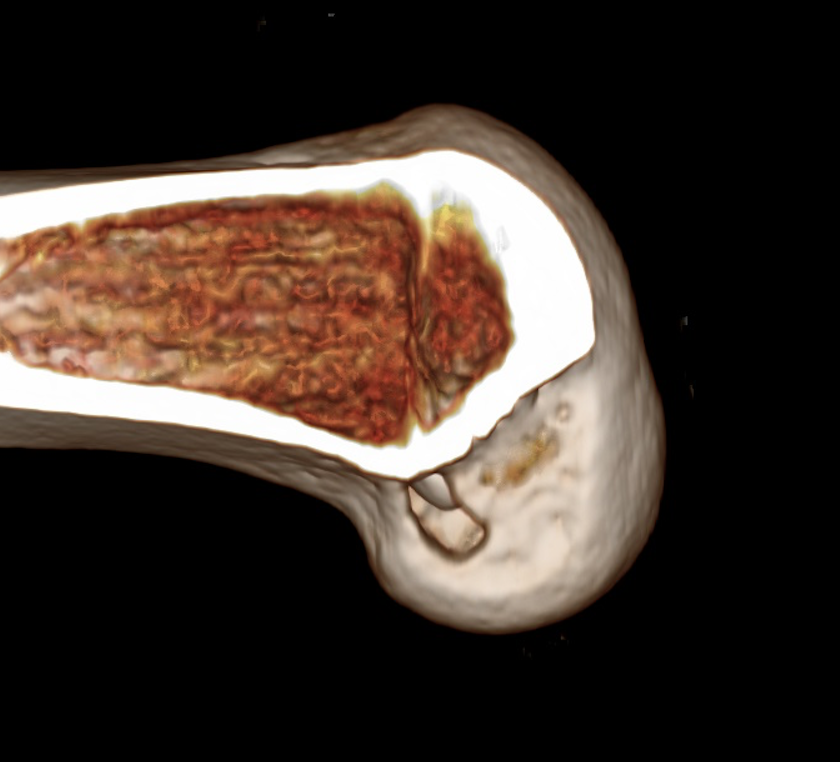

Supplement: S1 Dataset — Data required to calculate and replicate all the figures and tables. (ZIP) [file pone.0215778.s003.zip › 3DCT PLOS ONE/1/femur.png]

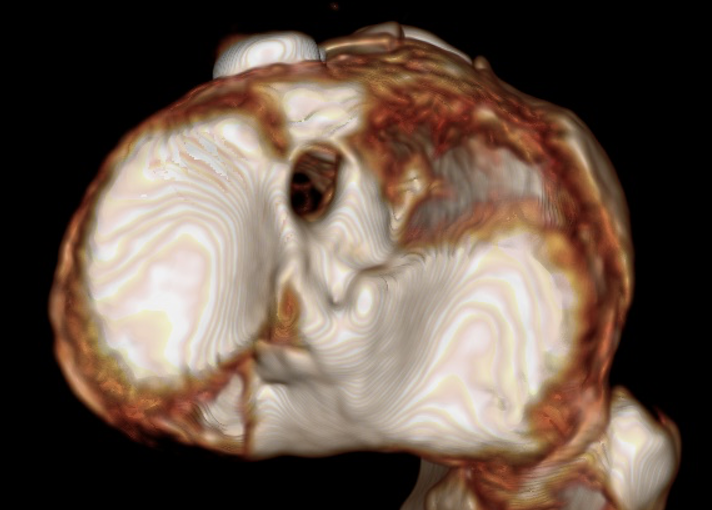

Supplement: S1 Dataset — Data required to calculate and replicate all the figures and tables. (ZIP) [file pone.0215778.s003.zip › 3DCT PLOS ONE/10/tibia.png]

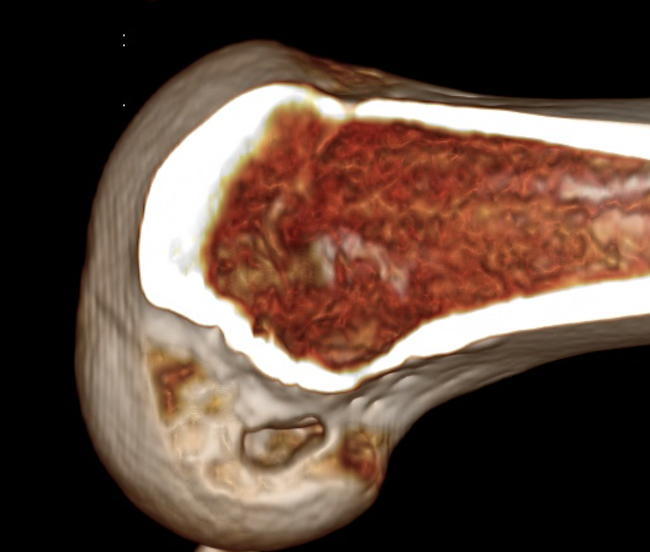

Supplement: S1 Dataset — Data required to calculate and replicate all the figures and tables. (ZIP) [file pone.0215778.s003.zip › 3DCT PLOS ONE/10/femur.png]

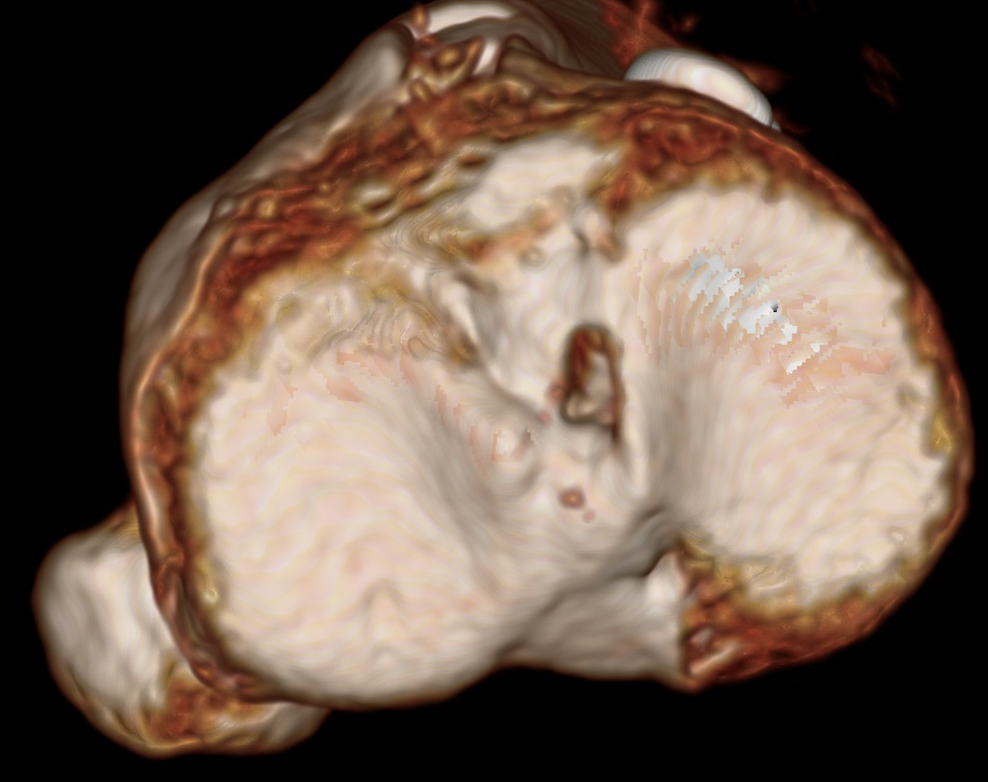

Supplement: S1 Dataset — Data required to calculate and replicate all the figures and tables. (ZIP) [file pone.0215778.s003.zip › 3DCT PLOS ONE/19/tibia.png]

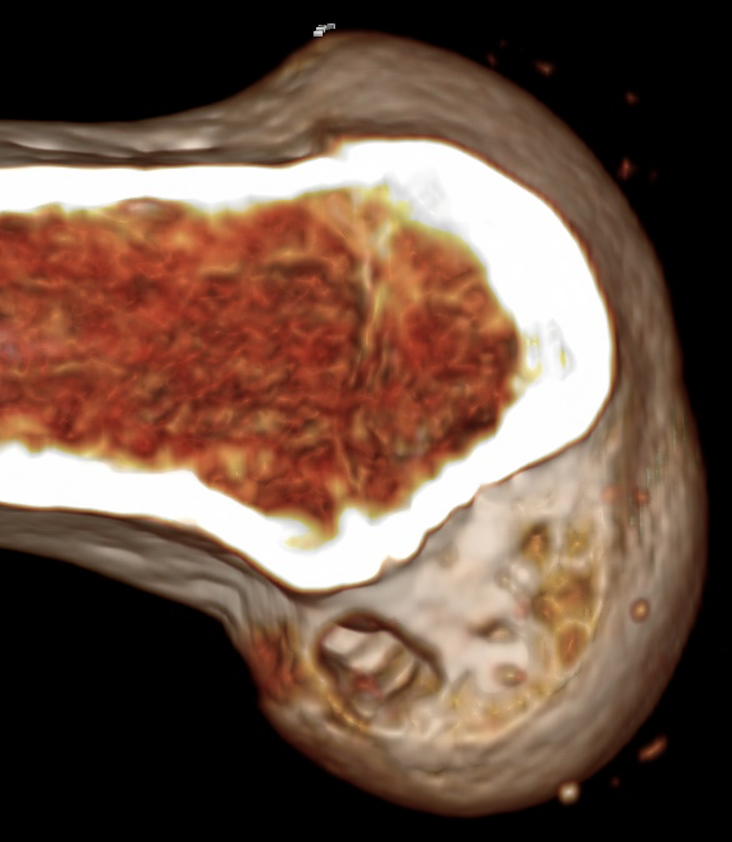

Supplement: S1 Dataset — Data required to calculate and replicate all the figures and tables. (ZIP) [file pone.0215778.s003.zip › 3DCT PLOS ONE/19/femur.png]

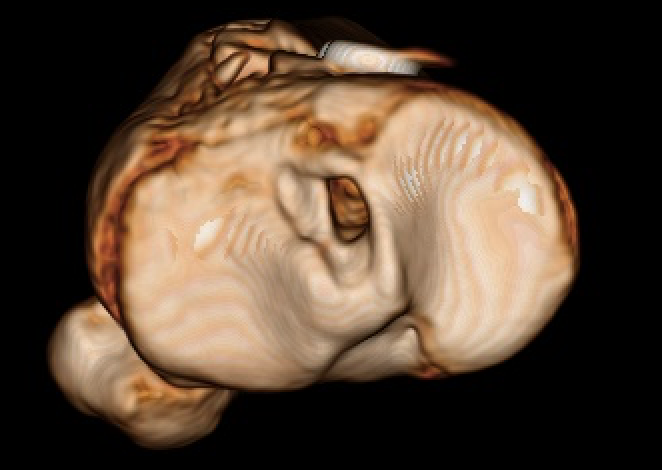

Supplement: S1 Dataset — Data required to calculate and replicate all the figures and tables. (ZIP) [file pone.0215778.s003.zip › 3DCT PLOS ONE/26/tibia.png]

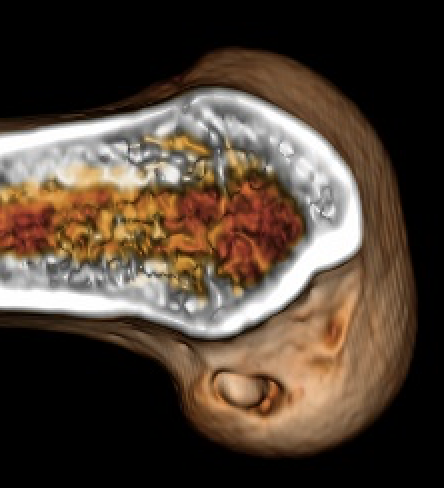

Supplement: S1 Dataset — Data required to calculate and replicate all the figures and tables. (ZIP) [file pone.0215778.s003.zip › 3DCT PLOS ONE/26/femur.png]

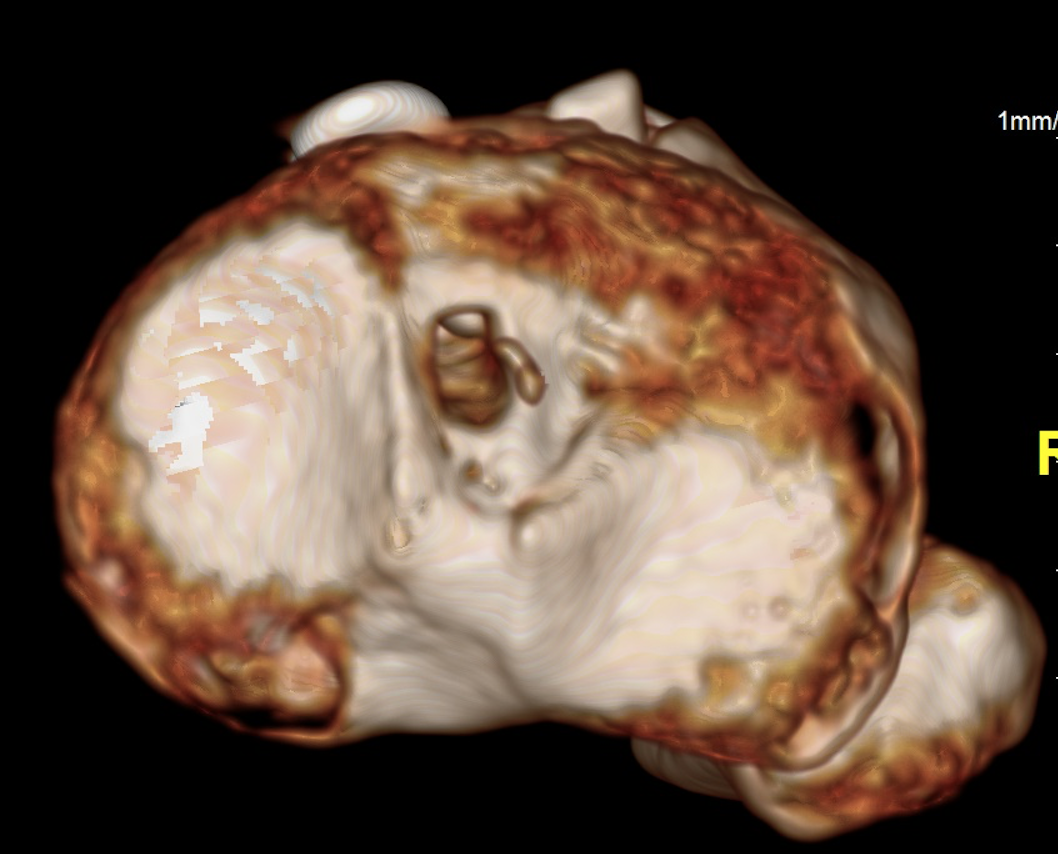

Supplement: S1 Dataset — Data required to calculate and replicate all the figures and tables. (ZIP) [file pone.0215778.s003.zip › 3DCT PLOS ONE/8/tibia.png]

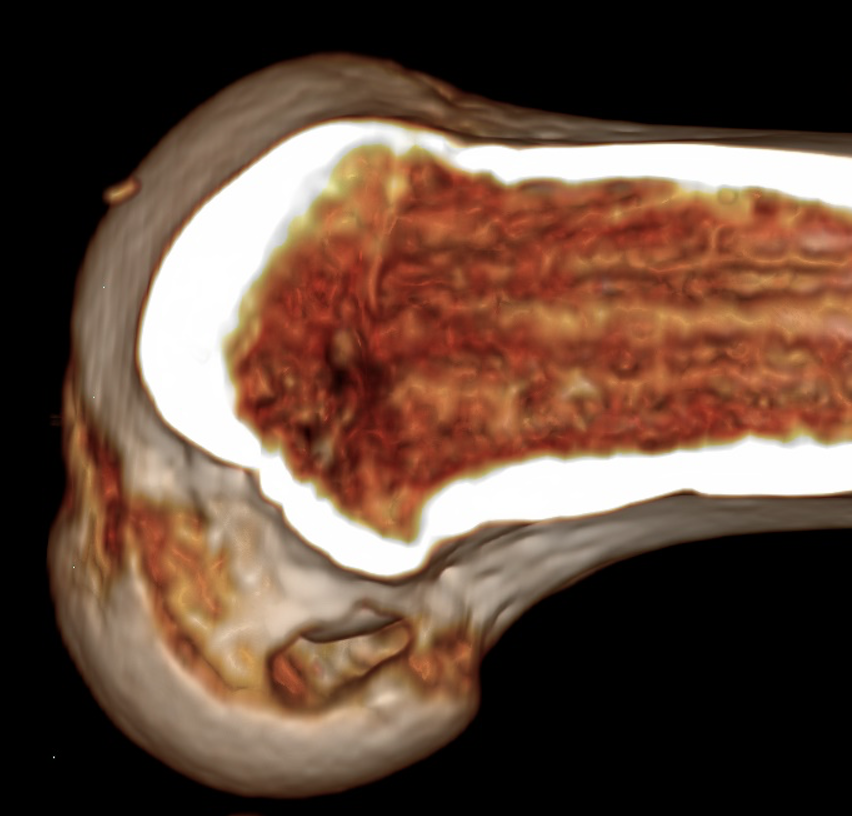

Supplement: S1 Dataset — Data required to calculate and replicate all the figures and tables. (ZIP) [file pone.0215778.s003.zip › 3DCT PLOS ONE/8/femur.png]

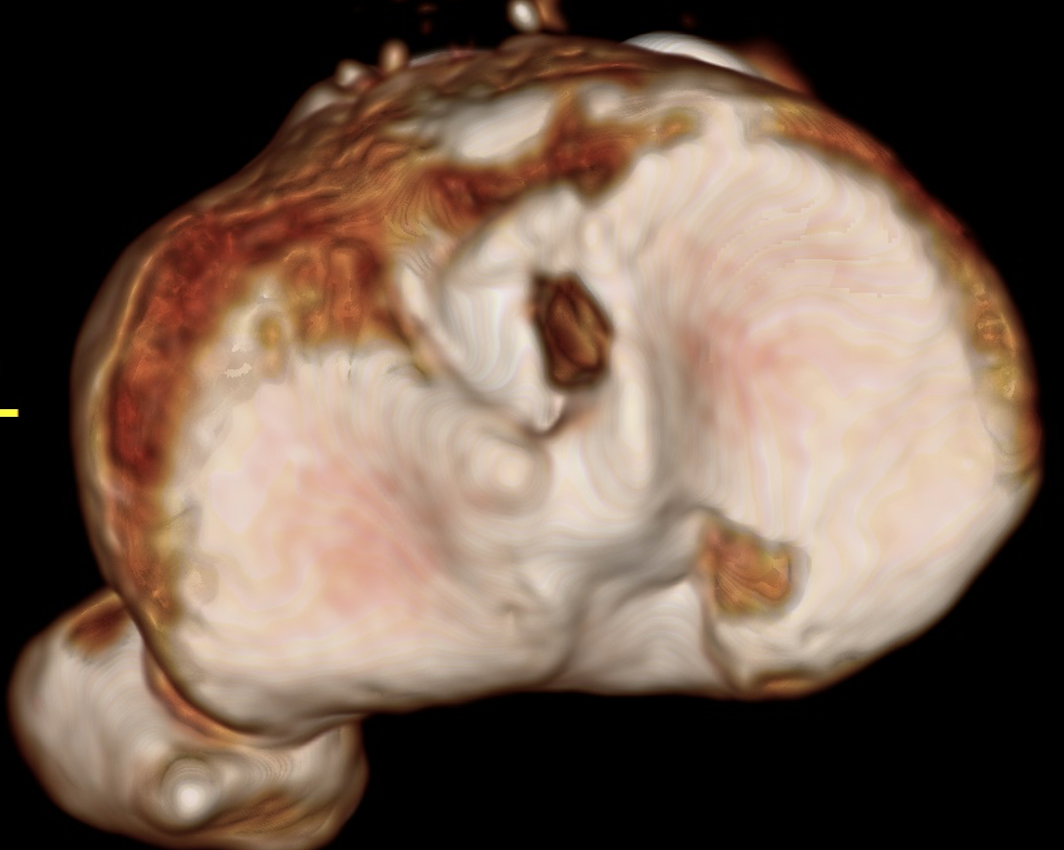

Supplement: S1 Dataset — Data required to calculate and replicate all the figures and tables. (ZIP) [file pone.0215778.s003.zip › 3DCT PLOS ONE/21/tibia.png]

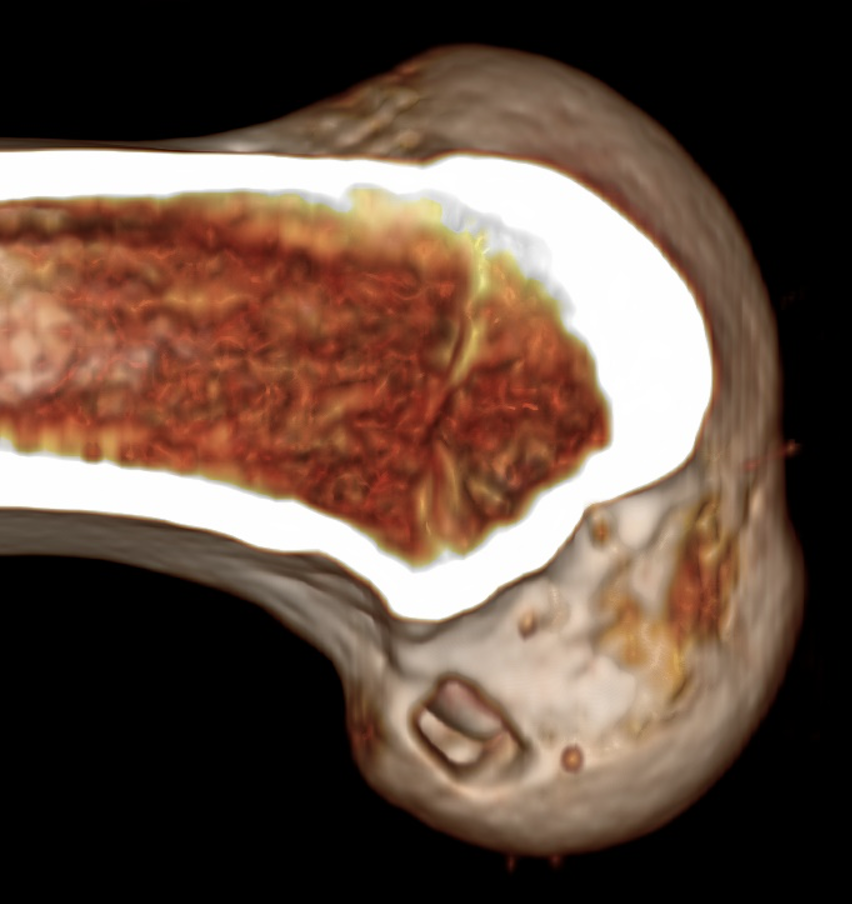

Supplement: S1 Dataset — Data required to calculate and replicate all the figures and tables. (ZIP) [file pone.0215778.s003.zip › 3DCT PLOS ONE/21/femur.png]

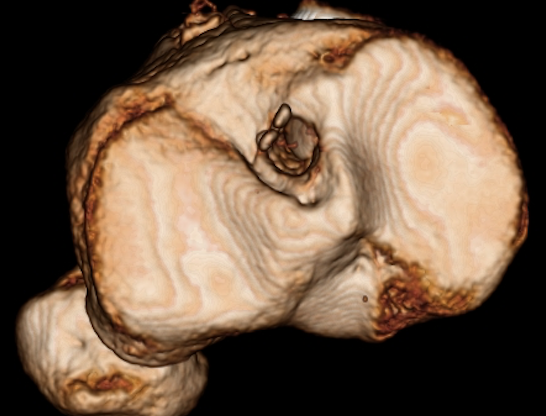

Supplement: S1 Dataset — Data required to calculate and replicate all the figures and tables. (ZIP) [file pone.0215778.s003.zip › 3DCT PLOS ONE/38/tibia.png]

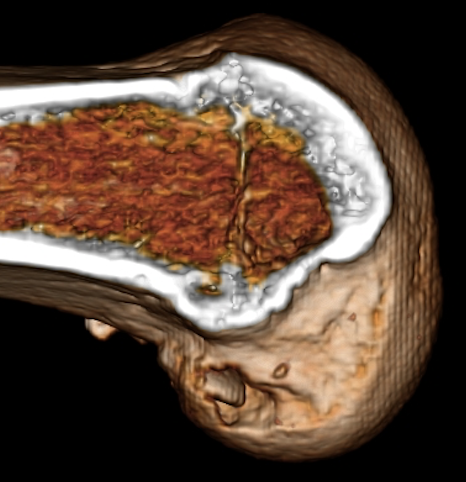

Supplement: S1 Dataset — Data required to calculate and replicate all the figures and tables. (ZIP) [file pone.0215778.s003.zip › 3DCT PLOS ONE/38/femur.png]

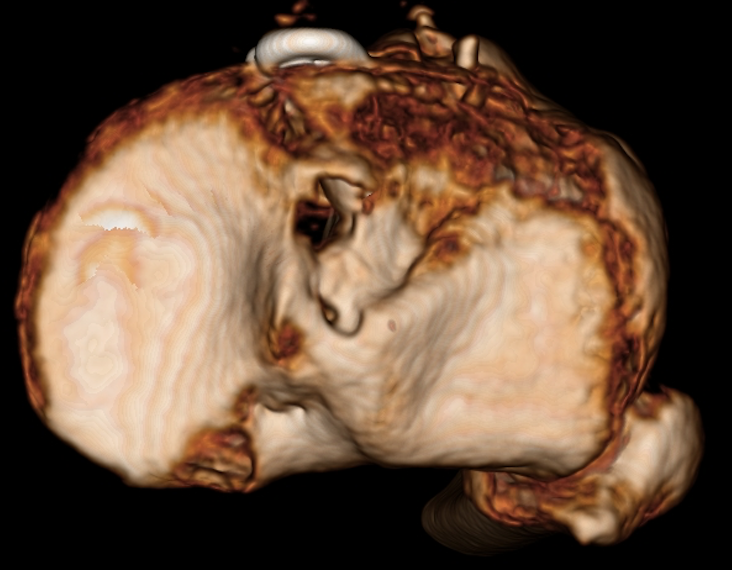

Supplement: S1 Dataset — Data required to calculate and replicate all the figures and tables. (ZIP) [file pone.0215778.s003.zip › 3DCT PLOS ONE/36/tibia.png]

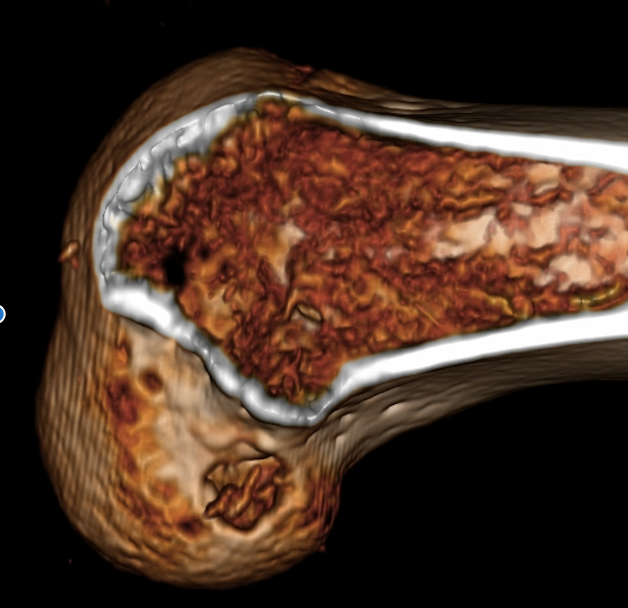

Supplement: S1 Dataset — Data required to calculate and replicate all the figures and tables. (ZIP) [file pone.0215778.s003.zip › 3DCT PLOS ONE/36/femur.png]

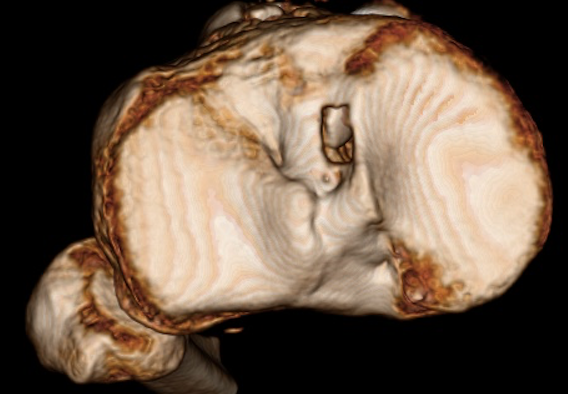

Supplement: S1 Dataset — Data required to calculate and replicate all the figures and tables. (ZIP) [file pone.0215778.s003.zip › 3DCT PLOS ONE/31/tibia.png]

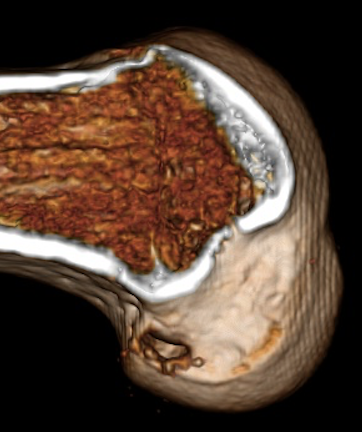

Supplement: S1 Dataset — Data required to calculate and replicate all the figures and tables. (ZIP) [file pone.0215778.s003.zip › 3DCT PLOS ONE/31/femur.png]

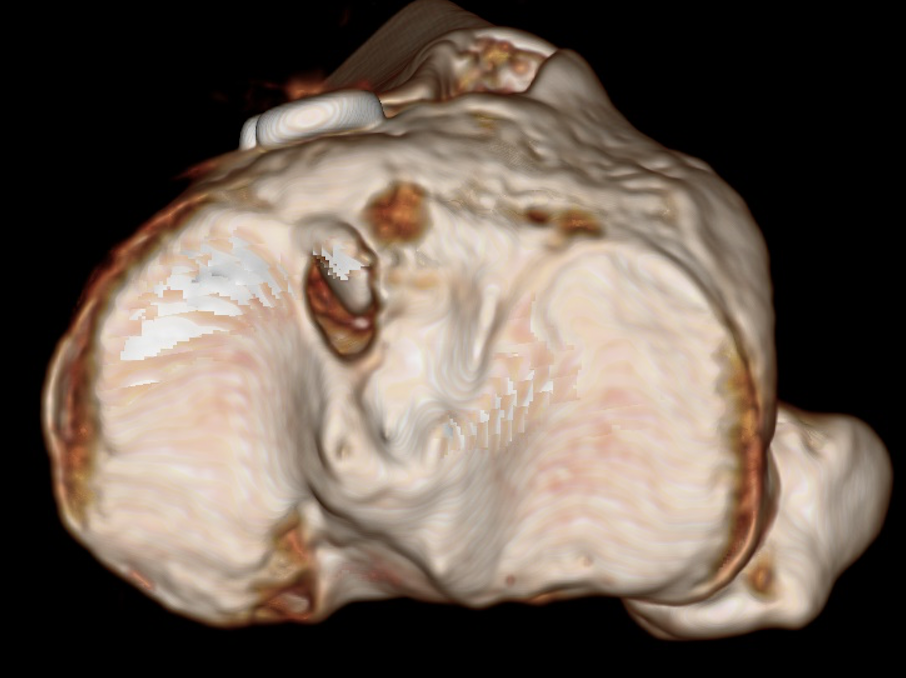

Supplement: S1 Dataset — Data required to calculate and replicate all the figures and tables. (ZIP) [file pone.0215778.s003.zip › 3DCT PLOS ONE/30/tibia.png]

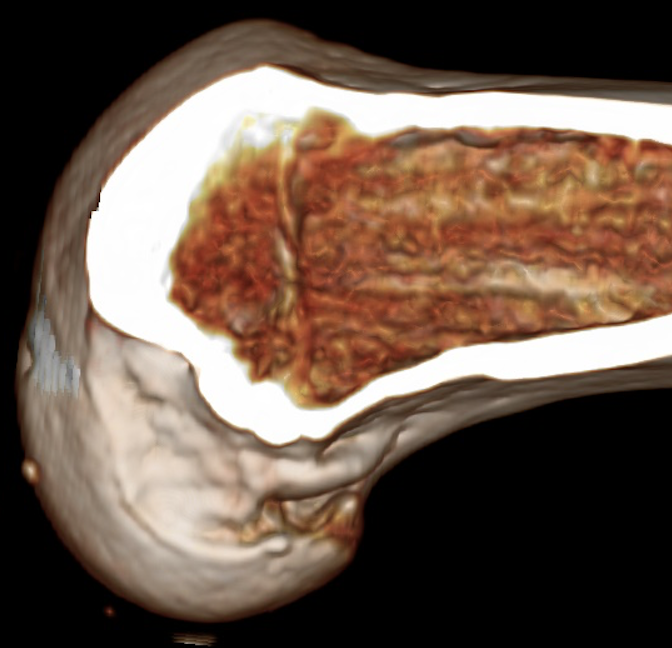

Supplement: S1 Dataset — Data required to calculate and replicate all the figures and tables. (ZIP) [file pone.0215778.s003.zip › 3DCT PLOS ONE/30/femur.png]

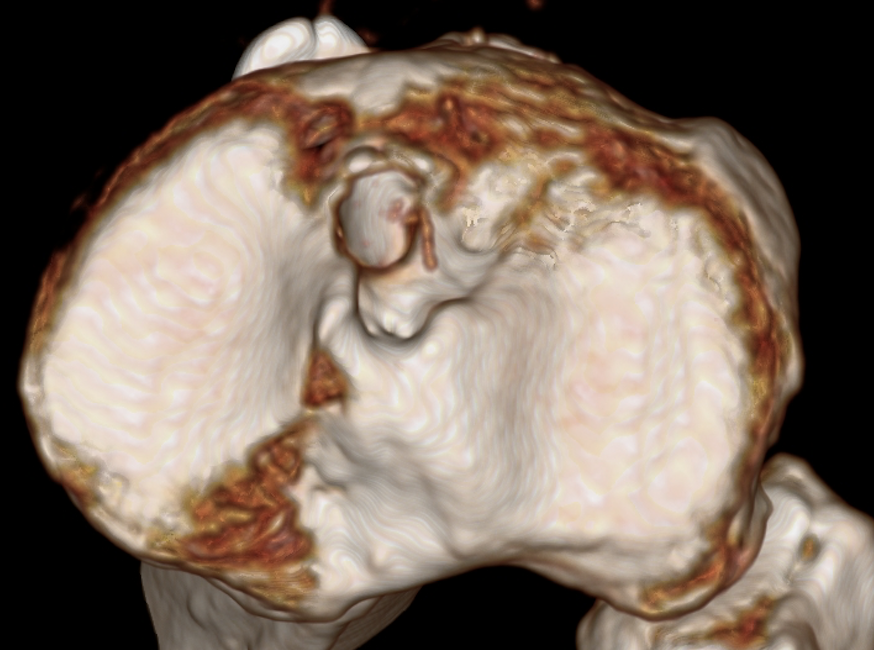

Supplement: S1 Dataset — Data required to calculate and replicate all the figures and tables. (ZIP) [file pone.0215778.s003.zip › 3DCT PLOS ONE/37/tibia.png]

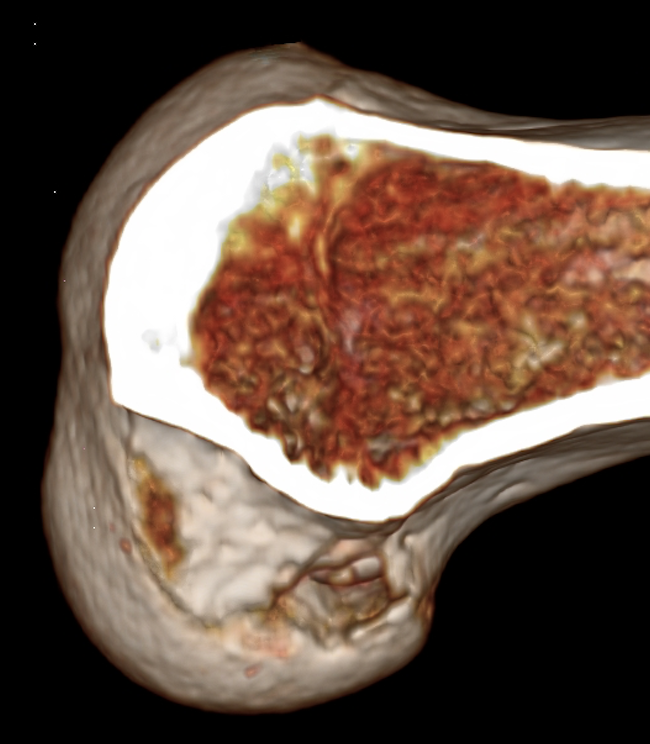

Supplement: S1 Dataset — Data required to calculate and replicate all the figures and tables. (ZIP) [file pone.0215778.s003.zip › 3DCT PLOS ONE/37/femur.png]

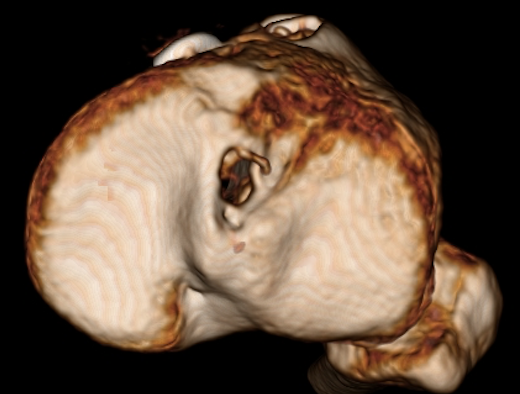

Supplement: S1 Dataset — Data required to calculate and replicate all the figures and tables. (ZIP) [file pone.0215778.s003.zip › 3DCT PLOS ONE/39/tibia.png]

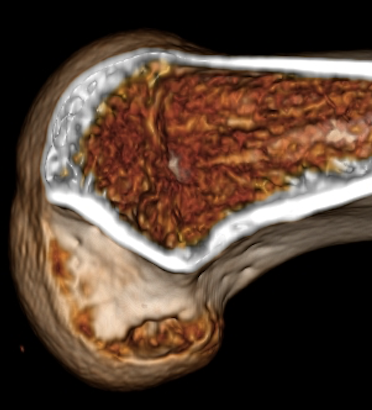

Supplement: S1 Dataset — Data required to calculate and replicate all the figures and tables. (ZIP) [file pone.0215778.s003.zip › 3DCT PLOS ONE/39/femur.png]

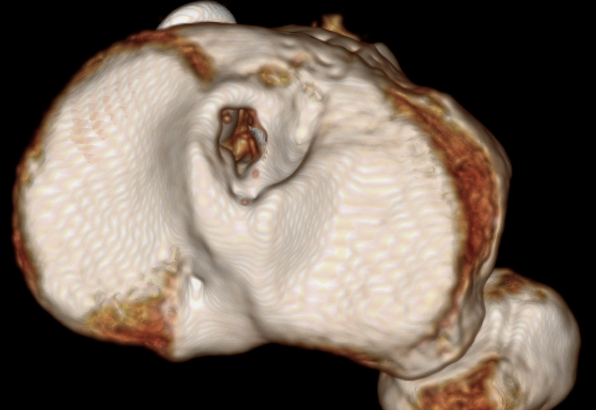

Supplement: S1 Dataset — Data required to calculate and replicate all the figures and tables. (ZIP) [file pone.0215778.s003.zip › 3DCT PLOS ONE/41/tibia.png]

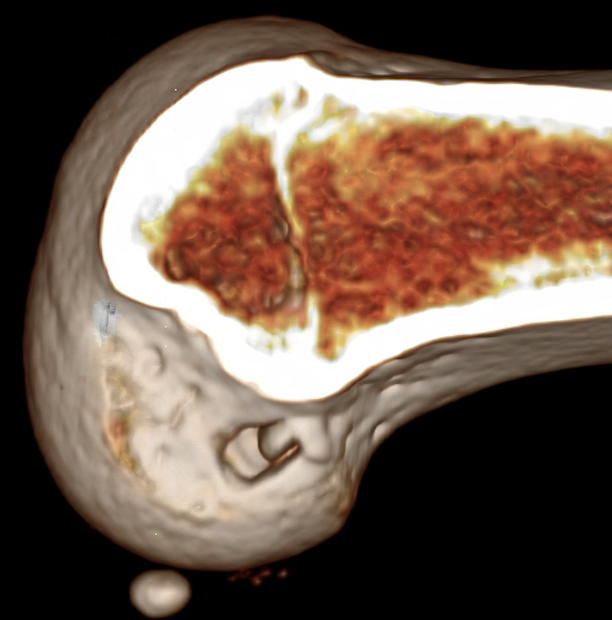

Supplement: S1 Dataset — Data required to calculate and replicate all the figures and tables. (ZIP) [file pone.0215778.s003.zip › 3DCT PLOS ONE/41/femur.png]

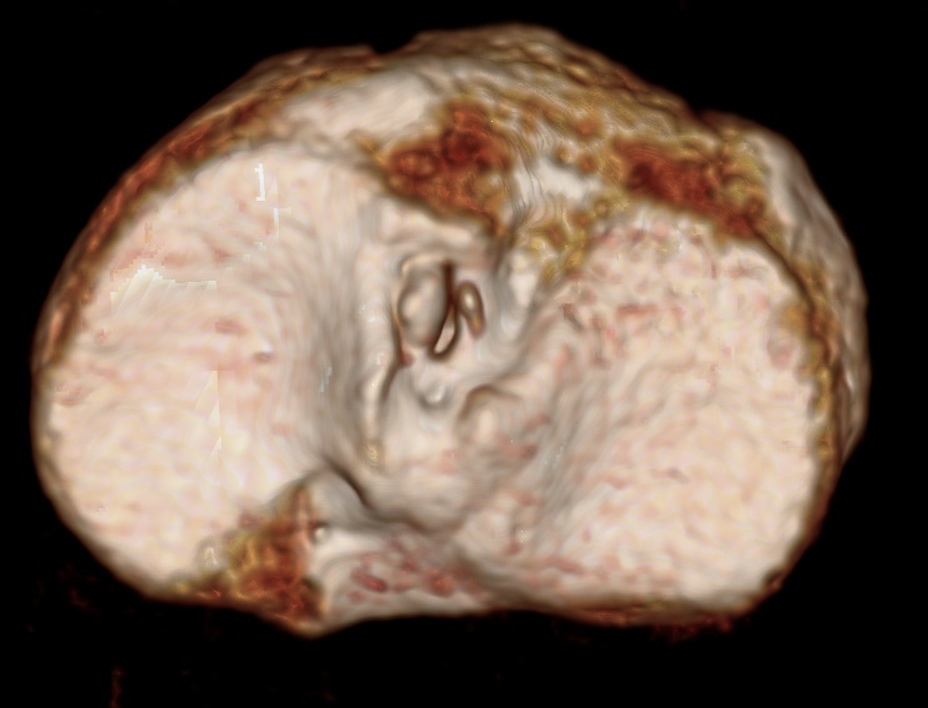

Supplement: S1 Dataset — Data required to calculate and replicate all the figures and tables. (ZIP) [file pone.0215778.s003.zip › 3DCT PLOS ONE/24/tibia.png]

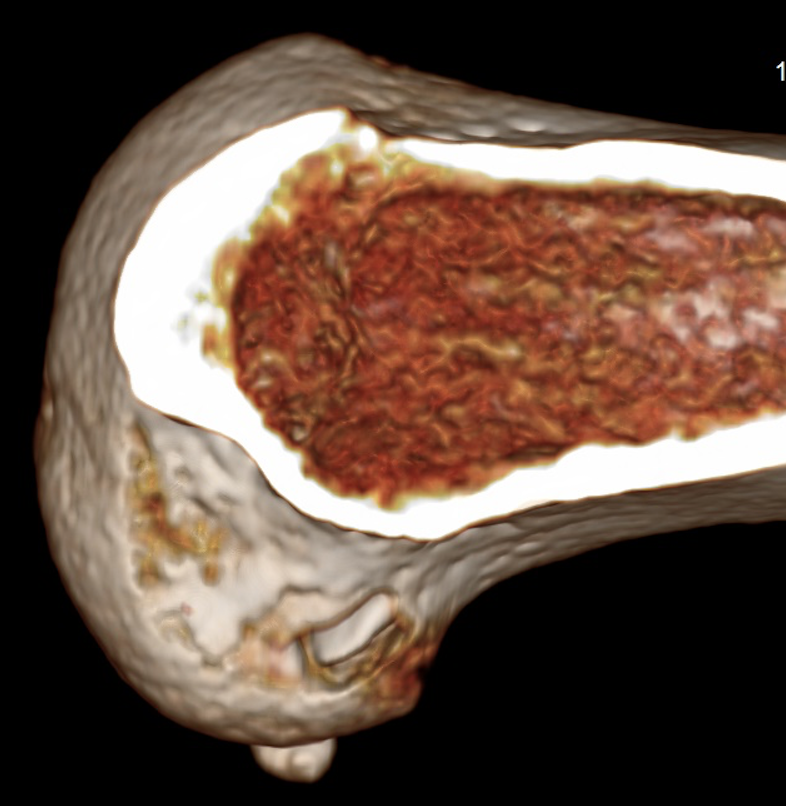

Supplement: S1 Dataset — Data required to calculate and replicate all the figures and tables. (ZIP) [file pone.0215778.s003.zip › 3DCT PLOS ONE/24/femur.png]

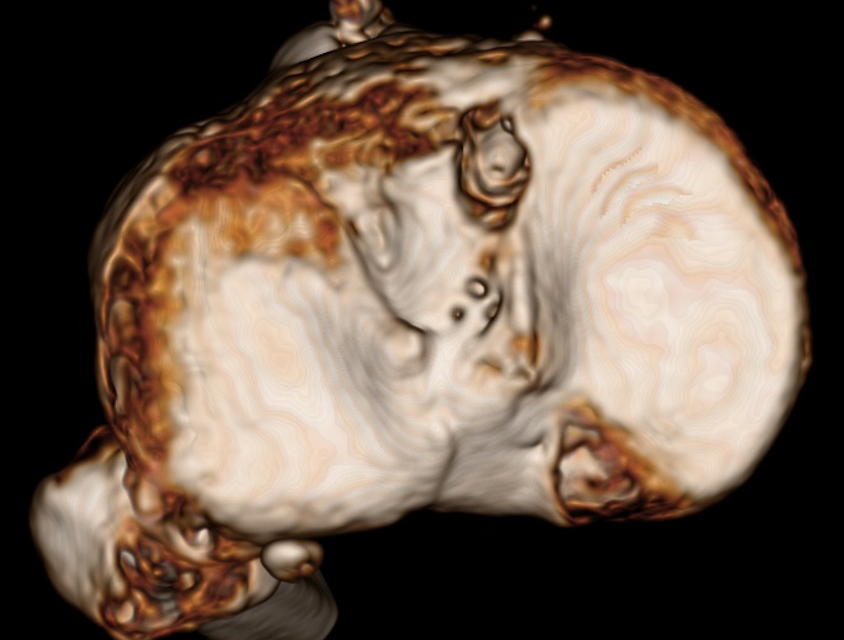

Supplement: S1 Dataset — Data required to calculate and replicate all the figures and tables. (ZIP) [file pone.0215778.s003.zip › 3DCT PLOS ONE/23/tibia.png]

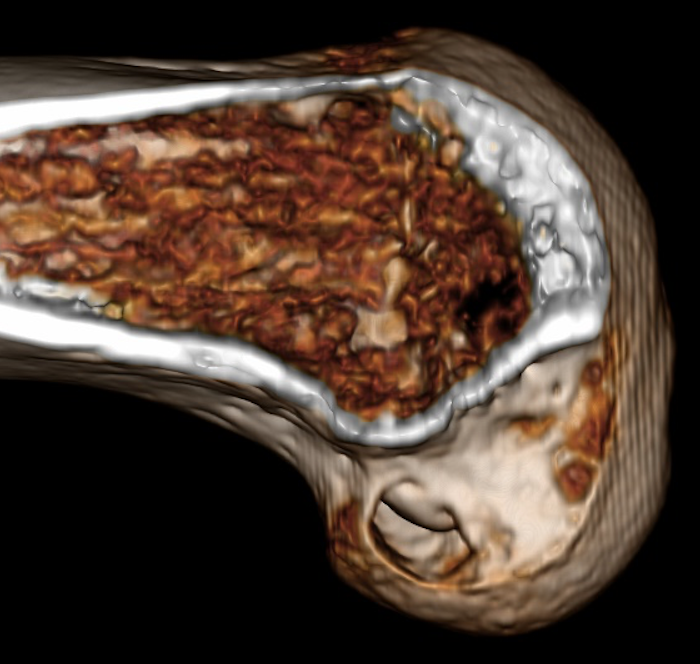

Supplement: S1 Dataset — Data required to calculate and replicate all the figures and tables. (ZIP) [file pone.0215778.s003.zip › 3DCT PLOS ONE/23/femur.png]

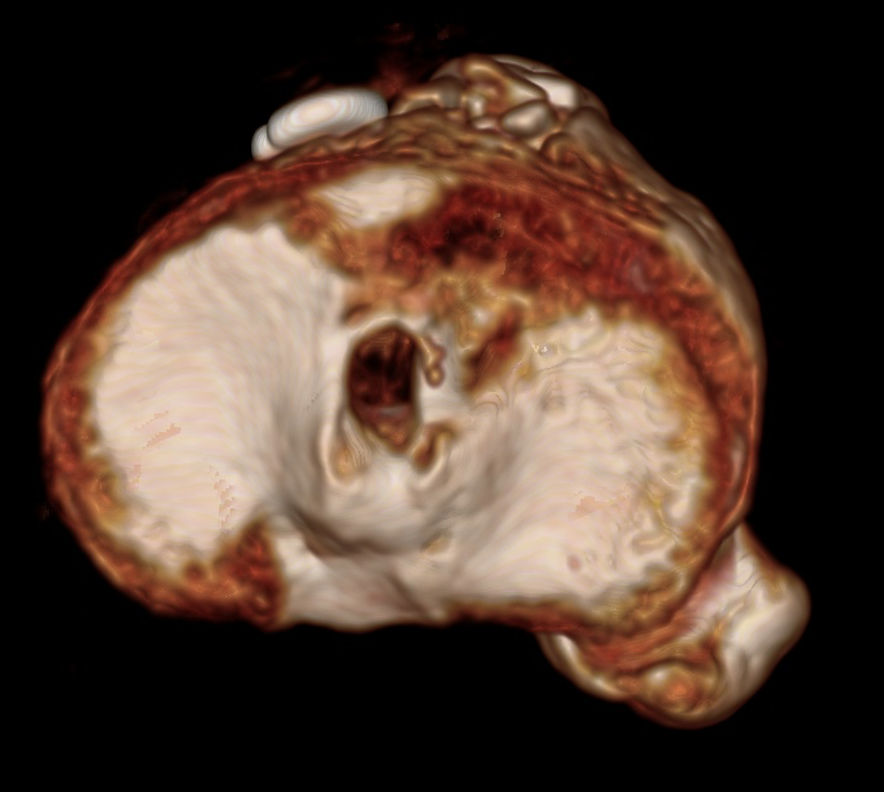

Supplement: S1 Dataset — Data required to calculate and replicate all the figures and tables. (ZIP) [file pone.0215778.s003.zip › 3DCT PLOS ONE/4/tibia.png]

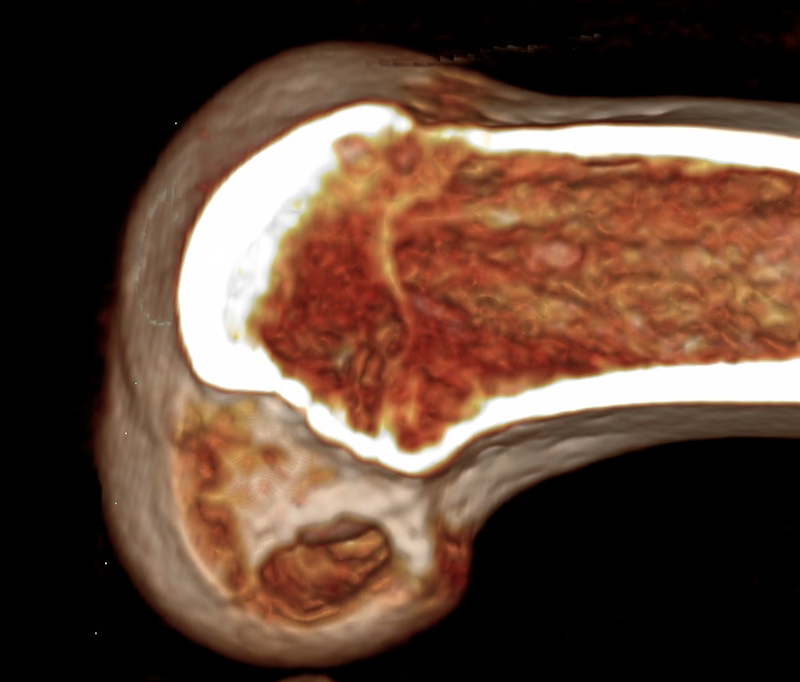

Supplement: S1 Dataset — Data required to calculate and replicate all the figures and tables. (ZIP) [file pone.0215778.s003.zip › 3DCT PLOS ONE/4/femur.png]

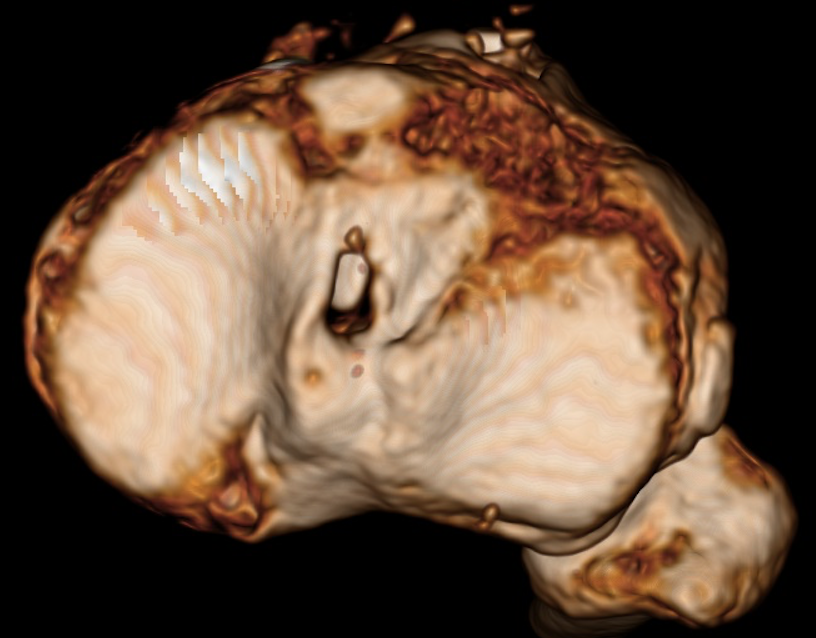

Supplement: S1 Dataset — Data required to calculate and replicate all the figures and tables. (ZIP) [file pone.0215778.s003.zip › 3DCT PLOS ONE/15/tibia.png]

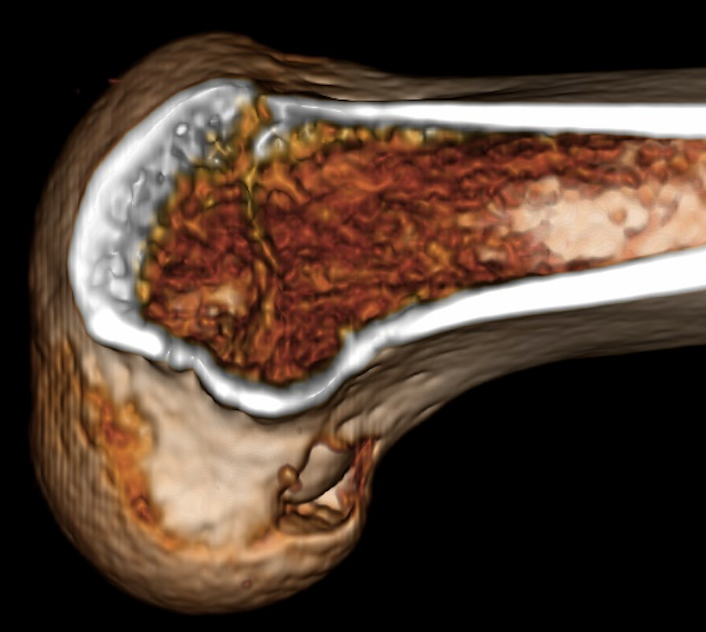

Supplement: S1 Dataset — Data required to calculate and replicate all the figures and tables. (ZIP) [file pone.0215778.s003.zip › 3DCT PLOS ONE/15/femur.png]

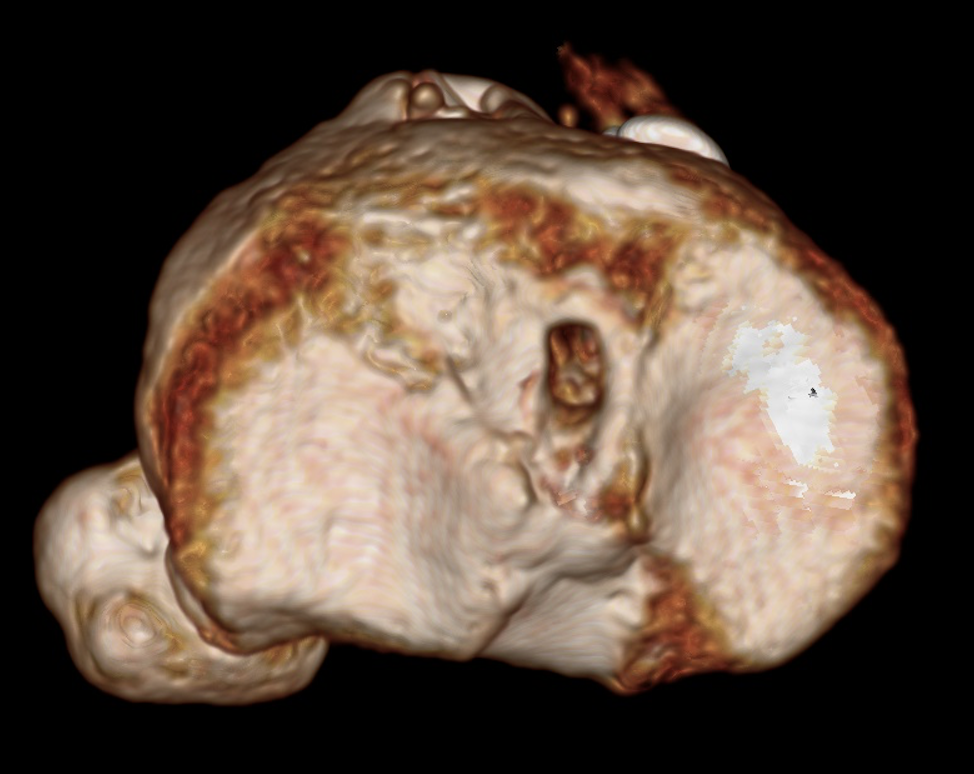

Supplement: S1 Dataset — Data required to calculate and replicate all the figures and tables. (ZIP) [file pone.0215778.s003.zip › 3DCT PLOS ONE/3/tibia.png]

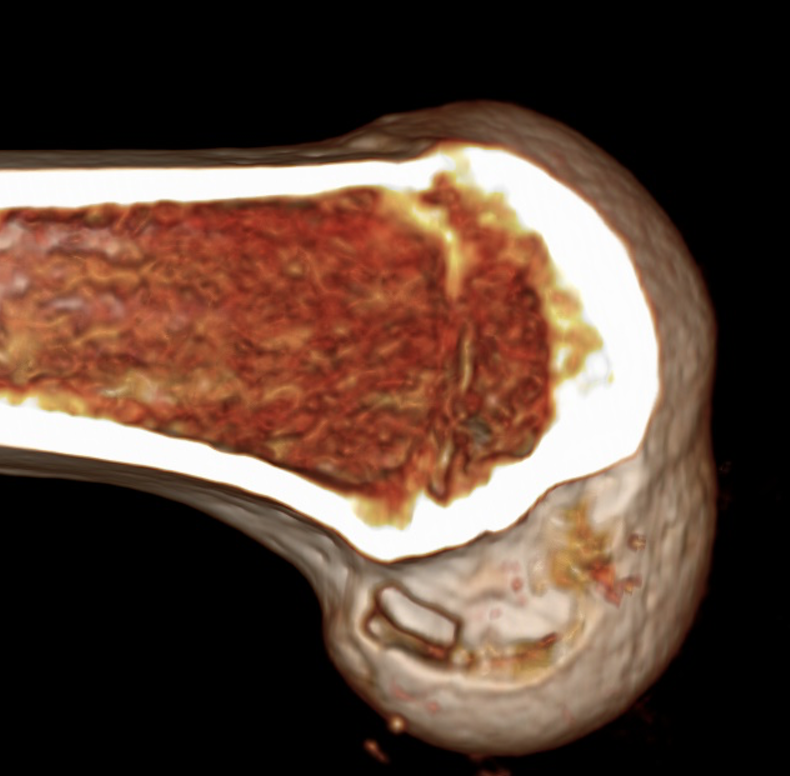

Supplement: S1 Dataset — Data required to calculate and replicate all the figures and tables. (ZIP) [file pone.0215778.s003.zip › 3DCT PLOS ONE/3/femur.png]

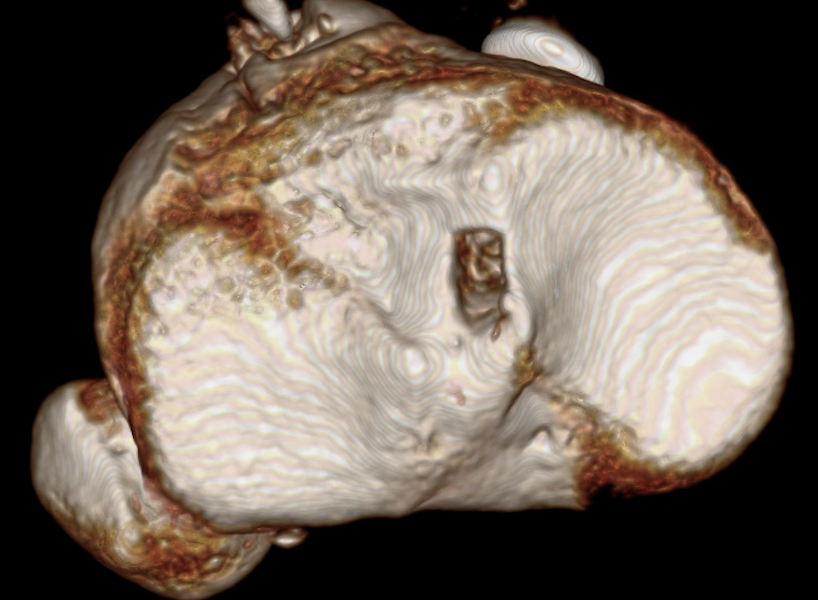

Supplement: S1 Dataset — Data required to calculate and replicate all the figures and tables. (ZIP) [file pone.0215778.s003.zip › 3DCT PLOS ONE/12/tibia.png]

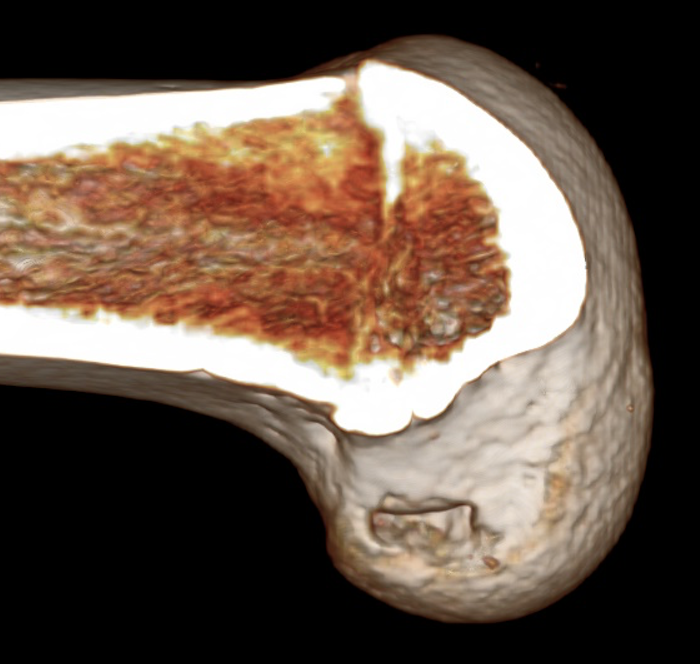

Supplement: S1 Dataset — Data required to calculate and replicate all the figures and tables. (ZIP) [file pone.0215778.s003.zip › 3DCT PLOS ONE/12/femur.png]

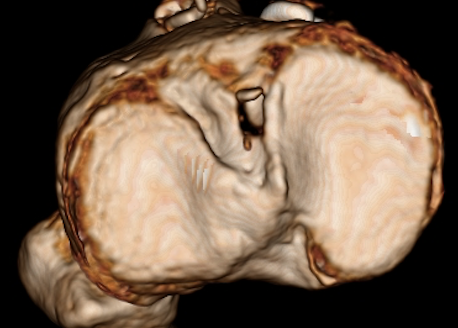

Supplement: S1 Dataset — Data required to calculate and replicate all the figures and tables. (ZIP) [file pone.0215778.s003.zip › 3DCT PLOS ONE/40/tibia.png]

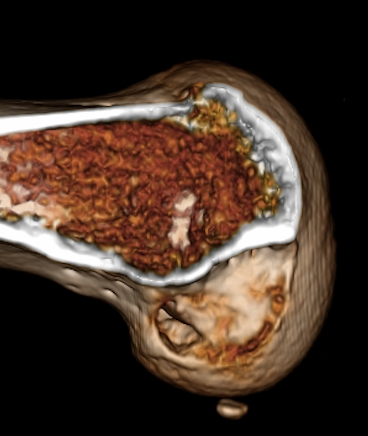

Supplement: S1 Dataset — Data required to calculate and replicate all the figures and tables. (ZIP) [file pone.0215778.s003.zip › 3DCT PLOS ONE/40/femur.png]

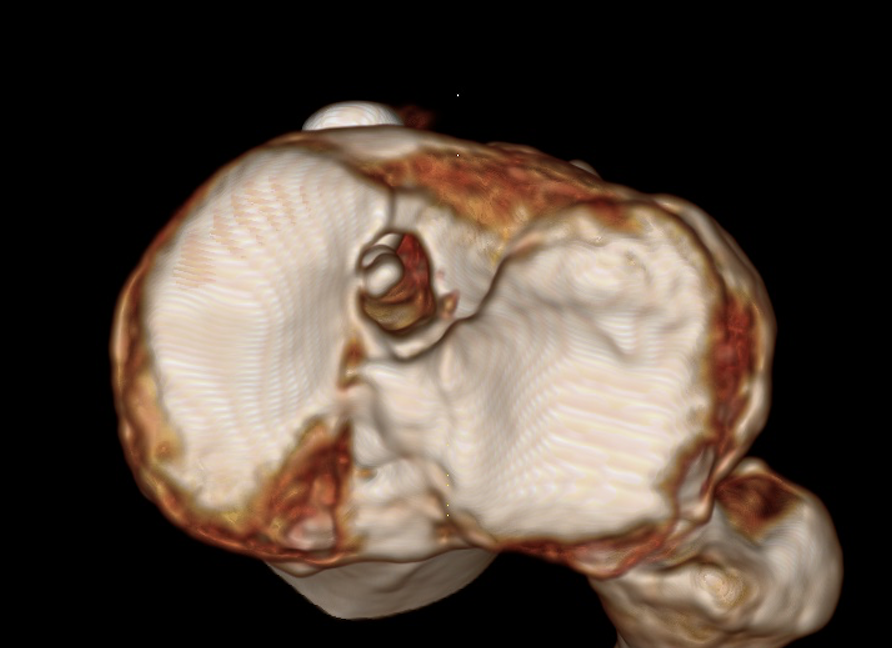

Supplement: S1 Dataset — Data required to calculate and replicate all the figures and tables. (ZIP) [file pone.0215778.s003.zip › 3DCT PLOS ONE/2/tibia.png]

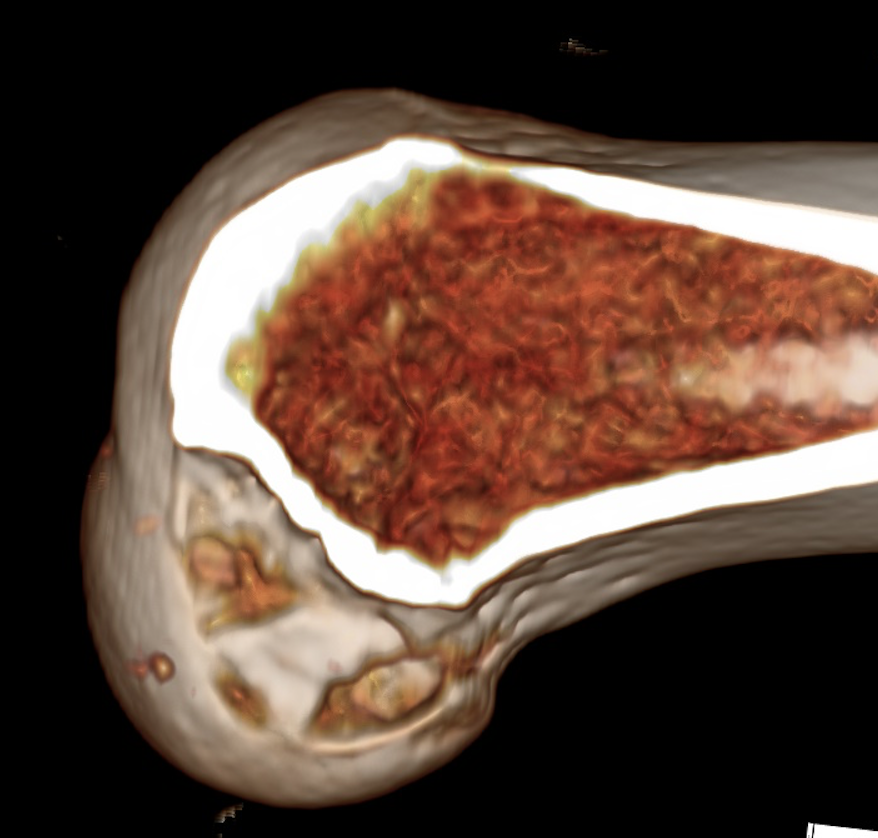

Supplement: S1 Dataset — Data required to calculate and replicate all the figures and tables. (ZIP) [file pone.0215778.s003.zip › 3DCT PLOS ONE/2/femur.png]

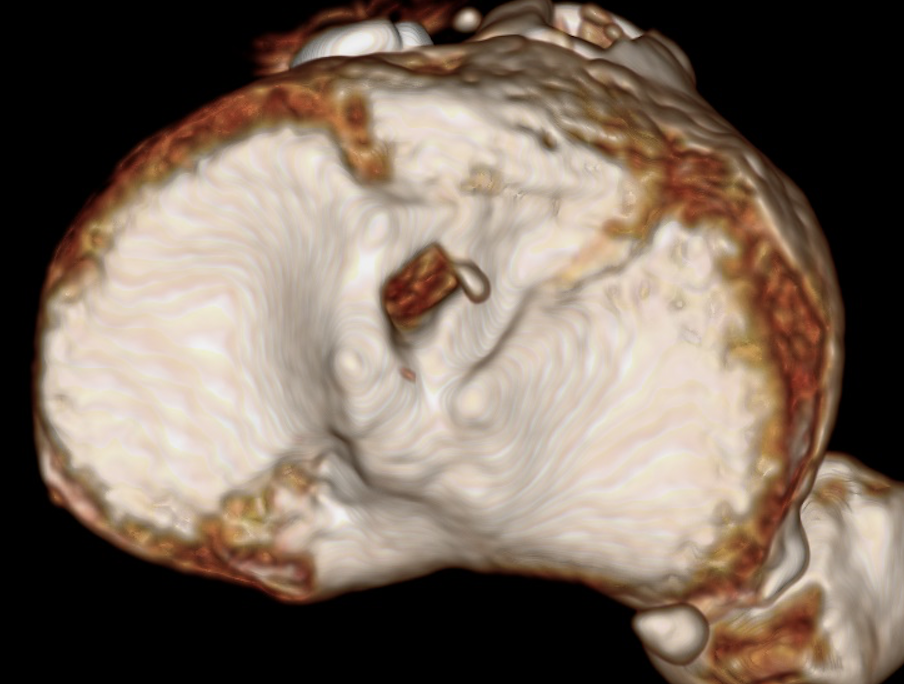

Supplement: S1 Dataset — Data required to calculate and replicate all the figures and tables. (ZIP) [file pone.0215778.s003.zip › 3DCT PLOS ONE/13/tibia.png]

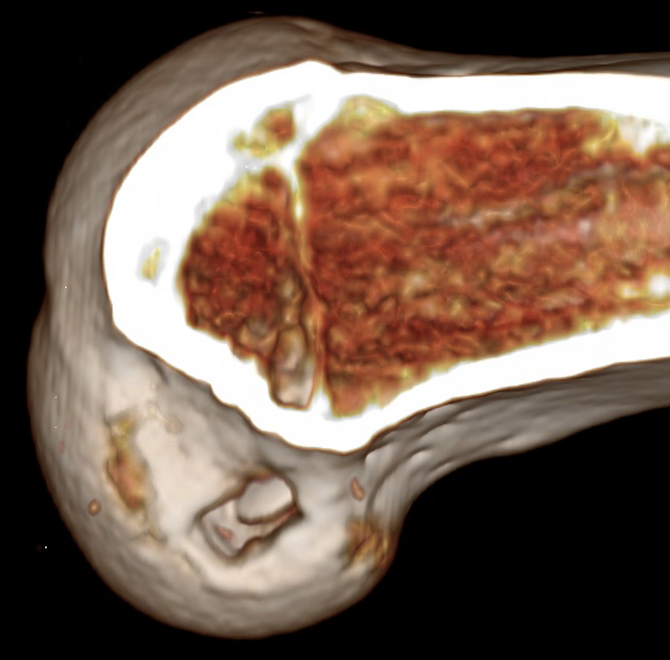

Supplement: S1 Dataset — Data required to calculate and replicate all the figures and tables. (ZIP) [file pone.0215778.s003.zip › 3DCT PLOS ONE/13/femur.png]

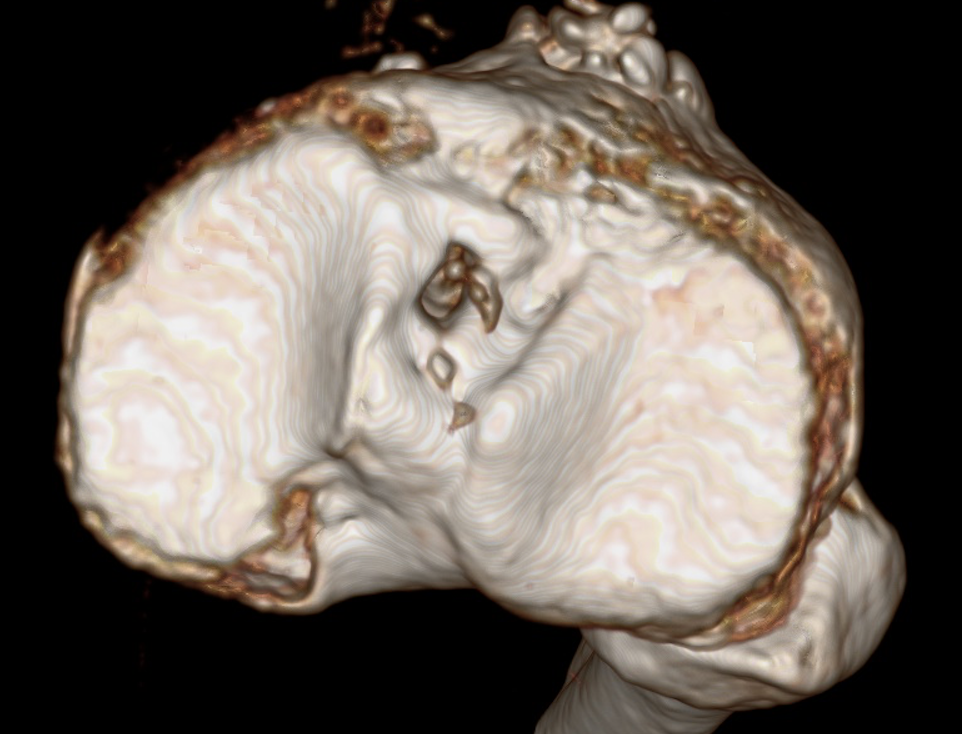

Supplement: S1 Dataset — Data required to calculate and replicate all the figures and tables. (ZIP) [file pone.0215778.s003.zip › 3DCT PLOS ONE/5/tibia.png]

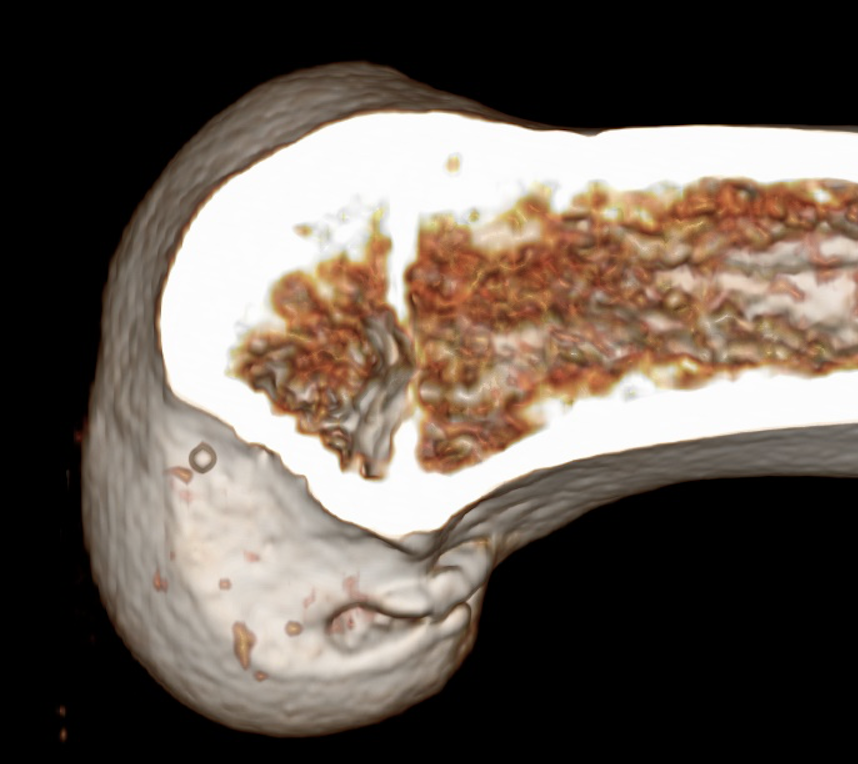

Supplement: S1 Dataset — Data required to calculate and replicate all the figures and tables. (ZIP) [file pone.0215778.s003.zip › 3DCT PLOS ONE/5/femur.png]

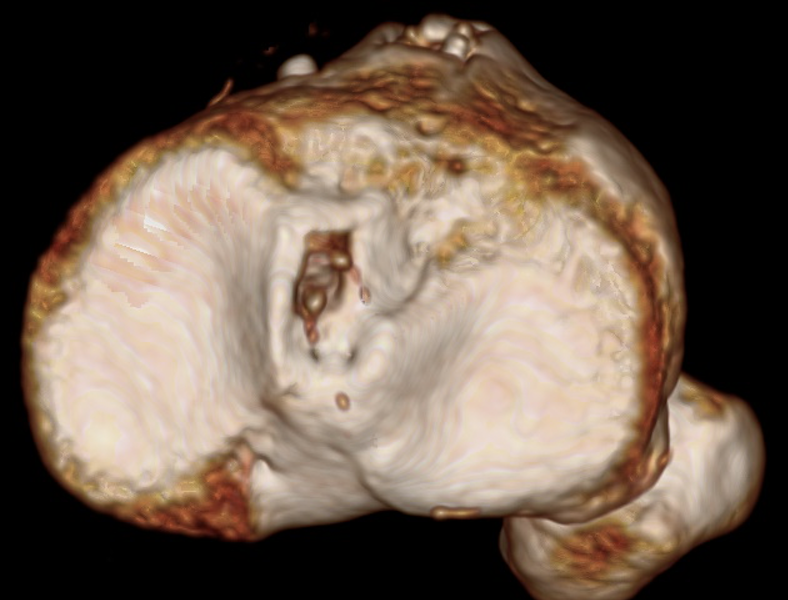

Supplement: S1 Dataset — Data required to calculate and replicate all the figures and tables. (ZIP) [file pone.0215778.s003.zip › 3DCT PLOS ONE/14/tibia.png]

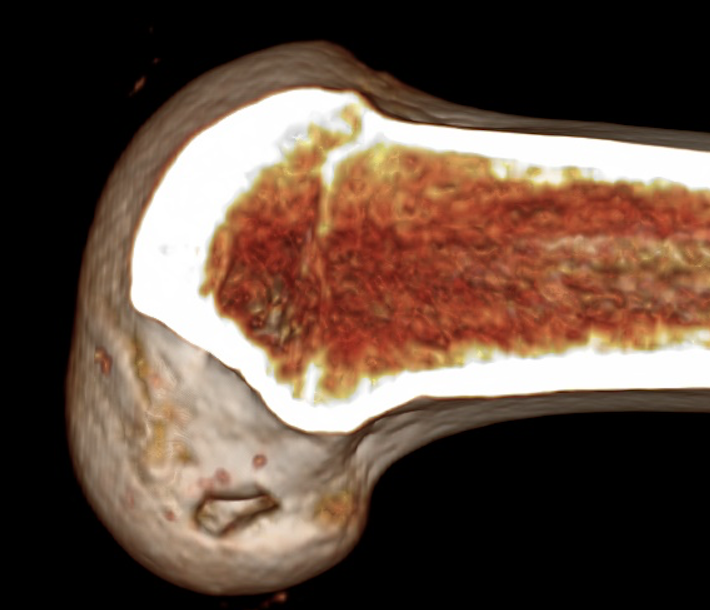

Supplement: S1 Dataset — Data required to calculate and replicate all the figures and tables. (ZIP) [file pone.0215778.s003.zip › 3DCT PLOS ONE/14/femur.png]

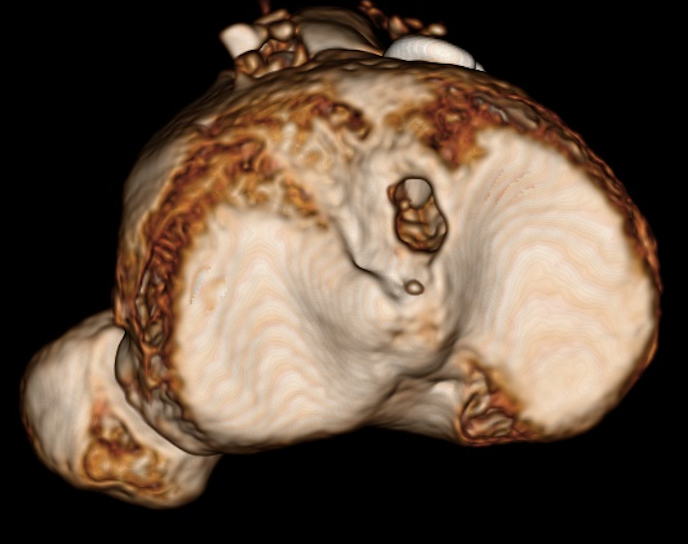

Supplement: S1 Dataset — Data required to calculate and replicate all the figures and tables. (ZIP) [file pone.0215778.s003.zip › 3DCT PLOS ONE/22/tibia.png]

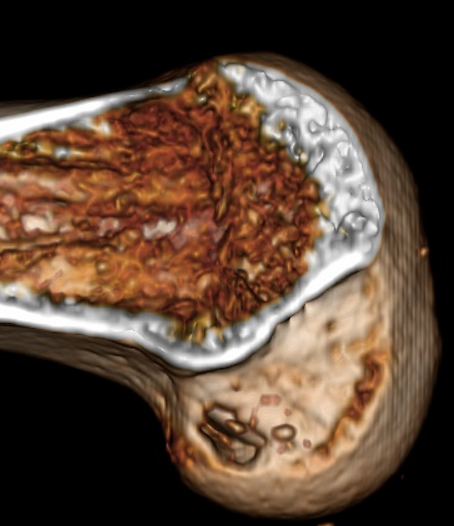

Supplement: S1 Dataset — Data required to calculate and replicate all the figures and tables. (ZIP) [file pone.0215778.s003.zip › 3DCT PLOS ONE/22/femur.png]

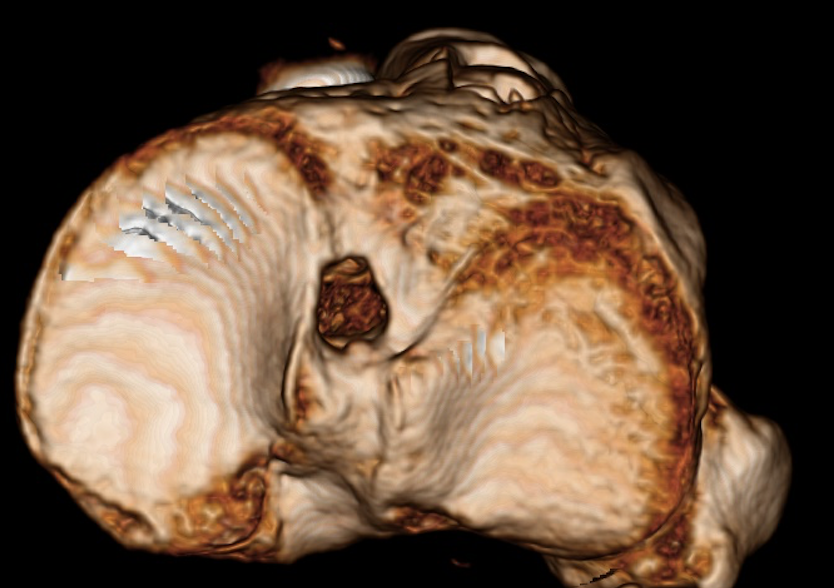

Supplement: S1 Dataset — Data required to calculate and replicate all the figures and tables. (ZIP) [file pone.0215778.s003.zip › 3DCT PLOS ONE/25/tibia.png]

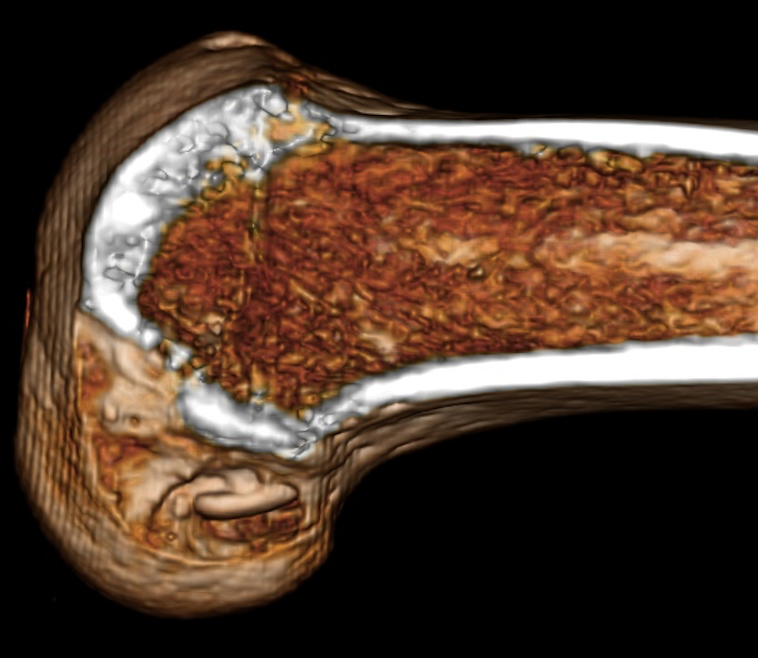

Supplement: S1 Dataset — Data required to calculate and replicate all the figures and tables. (ZIP) [file pone.0215778.s003.zip › 3DCT PLOS ONE/25/femur.png]
